# Homeostasis Imbalance of YY2 and YY1 Promotes Tumor Growth by Manipulating Ferroptosis

**DOI:** 10.1002/advs.202104836

**Published:** 2022-03-04

**Authors:** Yanjun Li, Juan Li, Zhuolin Li, Mankun Wei, Hezhao Zhao, Makoto Miyagishi, Shourong Wu, Vivi Kasim

**Affiliations:** ^1^ Key Laboratory of Biorheological Science and Technology Ministry of Education College of Bioengineering Chongqing University Chongqing 400044 China; ^2^ The 111 Project Laboratory of Biomechanics and Tissue Repair College of Bioengineering Chongqing University Chongqing 400044 China; ^3^ Department of Gastrointestinal Surgery Chongqing University Cancer Hospital Chongqing University Chongqing 400030 China; ^4^ Molecular Composite Physiology Research Group Health and Medical Research Institute National Institute of Advanced Industrial Science and Technology (AIST) Tsukuba 305‐8566 Japan; ^5^ Chongqing Key Laboratory of Translational Research for Cancer Metastasis and Individualized Treatment Chongqing University Cancer Hospital Chongqing University Chongqing 400030 China

**Keywords:** ferroptosis, lipid peroxidation, p53‐independent, Yin Yang 1, Yin Yang 2

## Abstract

Ferroptosis is a type of programmed cell death caused by disruption of redox homeostasis and is closely linked to amino acid metabolism. Yin Yang 2 (YY2) and its homolog Yin Yang 1 (YY1) are highly homologous, especially in their zinc‐finger domains. Furthermore, they share a consensus DNA binding motif. Increasing evidences have demonstrated the tumor suppressive effect of YY2, in contrast with the oncogenic YY1; however, little is known about the biological and pathological functions of YY2. Here, it is determined that YY2 induces tumor cell ferroptosis and subsequently suppresses tumorigenesis by inhibiting solute carrier family 7 member 11 (SLC7A11) transcription, leading to the decreased glutathione biosynthesis. Furthermore, YY2 and YY1 bind competitively to the same DNA binding site in the SLC7A11 promoter and antagonistically regulate tumor cell ferroptosis, thus suggesting the molecular mechanism underlying their opposite regulation on tumorigenesis. Moreover, mutations of YY2 zinc‐finger domains in clinical cancer patients abrogate YY2/SLC7A11 axis and tumor cell ferroptosis. Together, these results provide a new insight regarding the regulatory mechanism of ferroptosis, and a mechanistic explanation regarding the tumor suppressive effect of YY2. Finally, these findings demonstrate that homeostasis between YY1 and YY2 is crucial for maintaining redox homeostasis in tumor cells.

## Graphical abstract

Yin Yang 2 (YY2) is highly homologous with Yin Yang 1 (YY1), a famous oncogene; however, little is known about its functions. YY2 competes with YY1 to bind to SLC7A11 promoter and regulates it antagonistically, resulting in the opposite regulation on glutathione synthesis, ferroptosis, and tumor progression, indicating that YY2/YY1 homeostasis is crucial for maintaining redox homeostasis in tumors.

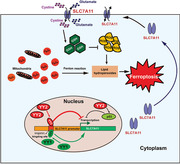

## 1 Introduction

Ferroptosis is an iron‐dependent, non‐apoptotic form of regulated cell death resulting from the disruption of redox homeostasis. It is characterized by excessive lipid peroxidation triggered by the accumulation of iron and reactive oxygen species (ROS), which in turn induces mitochondria shrinkage and cell death.^[^ ^1^, ^2^, ^3^ ^]^ Recent studies have shown a link between tumor cell amino acid metabolism and ferroptosis. Tumor cells obtain cysteine, the rate‐limiting precursor of glutathione (GSH) synthesis, through increasing system x_c_^−^ cystine‐glutamate antiporter‐mediated exchange of extracellular cystine with intracellular glutamate. Cystine is then reduced to cysteine, a component of GSH. Together with glutathione peroxidase 4, GSH reduces lipid peroxidases to lipid alcohols, thereby protecting tumor cells from oxidative stress.^[^ ^1^, ^4^, ^5^ ^]^ Importantly, blocking cystine uptake by inhibiting solute carrier family 7 member 11 (SLC7A11), the catalytic subunit of system x_c_^−^, could induce ferroptosis and suppress tumorigenesis.^[^ ^6^, ^7^, ^8^, ^9^ ^]^

Yin Yang 2 (YY2), a member of the Yin Yang (YY) family, is a C2H2 transcriptional factor that can activate or repress target genes.^[^ ^10^ ^]^ YY2 shares 86.4% amino acid sequence identity in the C‐terminal DNA binding zinc‐finger protein domains as well as consensus DNA binding motif (5′‐CGCCATnTT‐3′) with Yin Yang 1 (YY1), the first member of this family to be identified.^[^ ^11^ ^]^ YY1 plays important roles in various biological processes,^[^ ^10^, ^11^, ^12^, ^13^, ^14^, ^15^, ^16^ ^]^ and initially, YY2 was assumed to simply aid YY1 in regulating its target genes.^[^ ^11^ ^]^ Over time, increasing evidence has suggested that YY2 is not a redundant factor of YY1, but exerts its own specific function in maintaining embryonic stem cell growth, as well as in cardiovascular and nervous system development.^[^ ^17^, ^18^, ^19^, ^20^, ^21^ ^]^ However, unlike YY1, our knowledge about the biological function and regulatory mechanism of YY2 remains very limited.^[^ ^11^, ^17^, ^22^, ^23^ ^]^

YY1 is upregulated in various types of cancers, and is involved in the regulation of several hallmarks of cancer, such as tumor cell metabolic reprogramming, tumor angiogenesis, cell cycle regulation, and cell proliferation.^[^ ^14^, ^24^, ^25^, ^26^, ^27^, ^28^, ^29^, ^30^ ^]^ In contrast to oncogenic YY1, aberrantly low YY2 expression has been identified in various tumors, including breast, colon, and hepatocellular carcinoma.^[^ ^31^, ^32^ ^]^ In line with this evidence, YY2 suppression promotes tumor cell proliferation, while YY2 overexpression suppresses it, indicating that YY2 may exert an anti‐proliferative effect.^[^ ^17^, ^21^, ^32^ ^]^ However, the molecular mechanism underlying such tumor suppressive effect has not been completely understood. Furthermore, to our knowledge, there are no reports regarding the mechanism underlying the opposite effect of YY2 and YY1 in regulating tumorigenesis.

In this study, using transcriptomic analysis in addition to in vitro and in vivo experiments, we investigate a hitherto unrecognized function of YY2 in tumor cells ferroptosis and tumorigenesis through regulation of SLC7A11‐mediated GSH synthesis. Furthermore, we unravel the antagonistic function of YY2 and YY1 in regulating this pathway. Our findings highlight the importance of a balance between YY2 and YY1 for maintaining redox homeostasis and tumorigenesis.

## 2 Results

### 2.1 YY2 Suppresses Tumorigenesis

To elucidate the role of YY2 in tumorigenesis, we first constructed a YY2 overexpression vector, and confirmed its expression in HCT116 colorectal cancer cells, MHCC‐97H hepatocarcinoma cells and MCF‐7 breast cancer cells (Figure S1A, Supporting Information). We also constructed short hairpin RNA (shRNA) vectors targeting different sites on YY2. As the suppressive effects of shYY2‐1 and shYY2‐2 were similar (Figure S1B,C, Supporting Information), we chose shYY2‐1 for further experiments. Notably, neither the YY2 overexpression vector nor the shYY2 vectors affected the expression of YY1. The specificities of the anti‐YY2 and anti‐YY1 antibodies were confirmed by immunoprecipitation (Figure S1D, Supporting Information).

**Figure 1:**
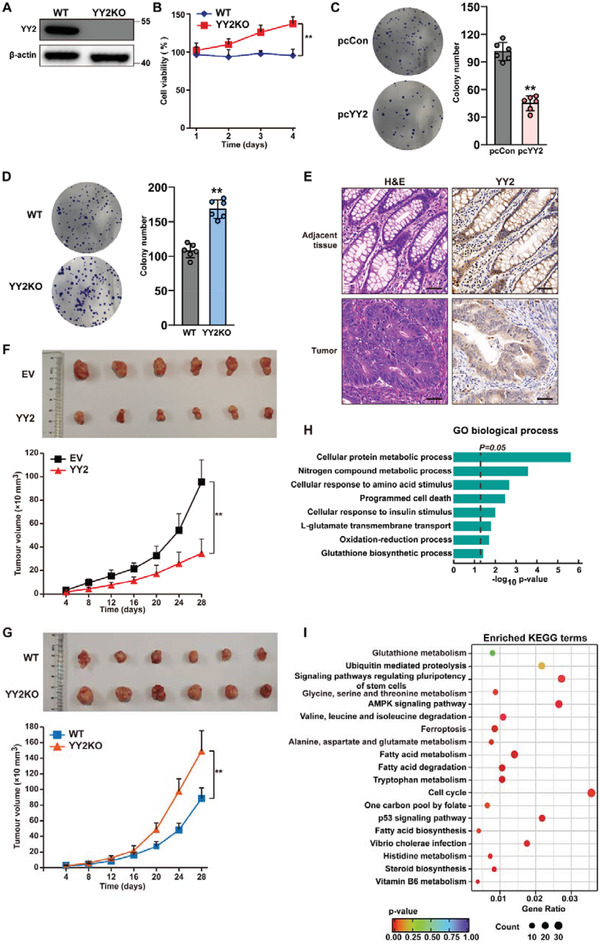
YY2 negatively regulates tumorigenesis. A) YY2 protein expression level in HCT116^YY2null^ cells, as determined using western blotting. B) Cell viability of HCT116^YY2null^ cells were measured at indicated time points. Colony formation potential of C) HCT116 cells overexpressing YY2 and D) YY2‐knocked out HCT116 cells. Representative images and quantification results (*n =* 6) are shown. E) YY2 expression level in clinical colon cancer tissue and the corresponding normal adjacent tissue, as analyzed using immunohistochemical staining. Scale bars, 50 *μ*m. Tumorigenesis potential of F) YY2‐overexpressed HCT116 and G) YY2‐knocked out HCT116 (HCT116^YY2null^) cells were examined in vivo by subcutaneous injection into Balb/c‐nu/nu mice (*n =* 6). Morphological images of the tumors generated at 28 days after injection (upper panels) and volume of the tumors formed at indicated time points (lower panels) are shown. H) Gene Ontology (GO) analysis from differentially expressed genes with *p*‐value < 0.05 (representing significantly altered genes; *n =* 3) in YY2‐overexpressing cells was performed using the database for annotation. The enriched GO biological processes were identified and listed according to their enrichment scores (−log10 (*p* value)). I) KEGGs enrichment from differentially expressed genes with *p*‐value < 0.05 (representing significantly enriched pathway). Wild‐type HCT116, cells transfected with pcCon or infected with empty lentivirus (EV) were used as controls. *β*‐actin was used as western blotting loading control. Quantification data are shown as mean ± SD (*n =* 3, unless further indicated). *p* values were calculated using two‐tailed unpaired Student's *t*‐test. pcCon: pcDNA3.1(+); YY2KO: HCT116^YY2null^ cells; ^**^ *p* < 0.01.

Next, we observed that YY2 overexpression significantly suppressed the viability of HCT116; whereas YY2 knockdown promoted their proliferation (Figure S2A, Supporting Information). Similar tendency was also observed in MHCC‐97H and MCF‐7 cells (Figure S2B, Supporting Information). To establish a YY2‐knocked out cell line, we used CRISPR‐Cas9 to target the sequence between positions 96 and 151 in the YY2 coding sequences (**Figure** **1**A; Figure S2C, Supporting Information). Similar to YY2 knockdown, YY2 knockout also did not affect YY1 expression (Figure S2D, Supporting Information). Viability was significantly higher in HCT116^YY2null^ cells compared to wild‐type cells (Figure 1B). Furthermore, overexpressing YY2 reduced the potential for colony formation by tumor cells, whereas knocking out YY2 enhanced it (Figure 1C,D). These results demonstrated that YY2 is a crucial suppressor of tumor cells proliferation. Moreover, its expression was conspicuously lower in colorectal cancer lesions compared to adjacent tissue (Figure 1E), suggesting a negative correlation between YY2 and tumor progression.

To determine whether YY2 could impact in vivo tumorigenesis, we used lentivirus to establish a stable YY2‐overexpressing HCT116 cell line (Figure S2E, Supporting Information). A xenograft experiment revealed that YY2 overexpression significantly retarded the growth of tumors formed by HCT116 cells transplanted subcutaneously into immunodeficient mice (Figure 1F); whereas tumors formed by HCT116^YY2null^ cells grew substantially faster (Figure 1G). Together, these results clearly demonstrated that YY2 is a tumor suppressor.

To elucidate the molecular mechanism underlying the tumor suppressive effect of YY2, we performed RNA sequencing (RNA‐Seq: GSE184138) to identify the genes differentially regulated by YY2. Gene Ontology (GO) and Kyoto Encyclopedia of Genes and Genomes (KEGG) analysis showed enrichment of genes related to “cellular protein metabolic process,” “cellular response to amino acid stimulus,” “programmed cell death,” “L‐glutamate transmembrane transport,” “oxidation‐reduction process,” and “glutathione biosynthesis process,” as well as “glutathione metabolism,” “ferroptosis,” and “alanine, aspartate, and glutamate metabolism” pathways in YY2‐overexpressing cells (Figure 1H,I). Given that ferroptosis is a programmed cell death triggered by the disruption of redox homeostasis, including aberrant GSH biosynthesis, we hypothesized that YY2 could regulate ferroptotic cell death. These results suggested that the tumor suppressive action of YY2 might be related to its involvement in cell death and ferroptosis.

### 2.2 YY2 Induces Ferroptotic Cell Death

**Figure 2:**
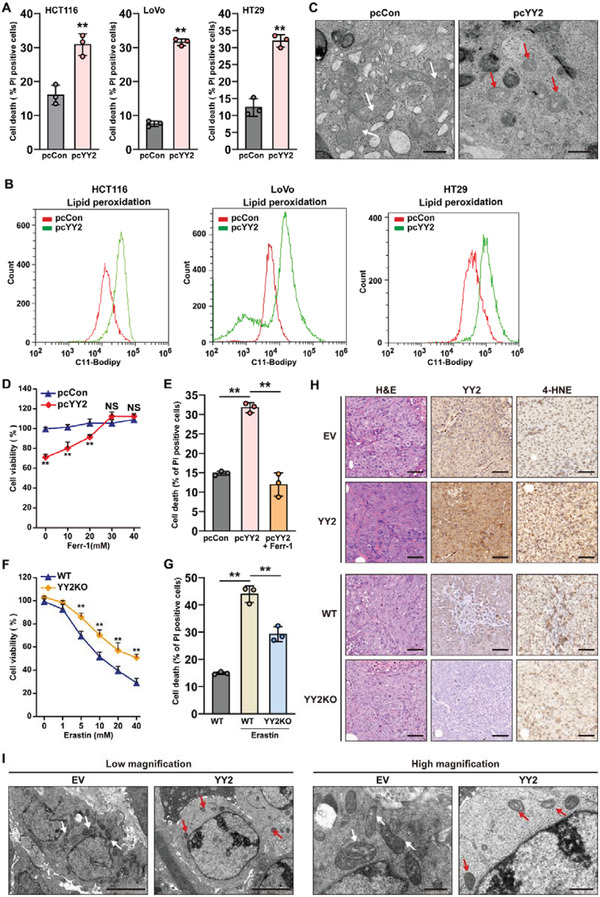
YY2 suppresses tumor cells viability by inducing ferroptosis. A) Cell death percentage of YY2‐overexpressed HCT116, and LoVo, HT29 cells, as analyzed using PI staining and flow cytometry. B) Lipid peroxidation in YY2‐overexpressed HCT116, LoVo, and HT29 cells, as assessed by C11‐BODIPY staining and flow cytometry. C) Transmission electron microscopy images of mitochondria in HCT116 cells overexpressing YY2. White arrows, mitochondria with obvious cristae; red arrows, shrunken mitochondria. Scale bars: 1 *μ*m. D) Cell viability of HCT116 cells overexpressing YY2 and treated with indicated concentration of ferrostatin‐1 for 36 h. E) Cell death percentage of HCT116 cells overexpressing YY2 and treated with 30 *μ* m ferrostatin‐1 for 24 h. F) Cell viability of HCT116^YY2null^ cells treated with indicated concentration of erastin for 36 h. G) Cell death percentage of HCT116^YY2null^ cells treated with 20 *μ* m erastin for 24 h. H) Immunohistochemical staining images against YY2 and 4‐HNE in the tissue section of xenografted tumors formed by HCT116 cells overexpressing YY2 and HCT116^YY2null^ cells. Scale bars, 50 *μ*m. I) Transmission electron microscopy images of the mitochondria in the xenograft tumors formed by HCT116 cells overexpressing YY2. White arrows: mitochondria with obvious cristae; red arrows: shrunken mitochondria. Scale bars: left, 5 *μ*m; right, 1 *μ*m. Wild‐type HCT116 cells, cells transfected with pcCon or infected with empty lentivirus (EV) were used as controls. Quantification data are shown as mean ± SD (*n =* 3). *p* values were calculated using two‐tailed unpaired Student's *t*‐test. One‐way ANOVA analyses were performed when more than two groups were compared. pcCon: pcDNA3.1(+); YY2KO: HCT116^YY2null^ cells; Ferr‐1: ferrostatin 1; ^**^ *p* < 0.01; NS: not significant.

Based on RNA‐Seq results, we further examined whether YY2 affected cell death and lipid peroxidation, an initial step in ferroptosis, using HCT116, LoVo, and HT29 colorectal cancer cells. YY2 overexpression robustly increased the percentage of propidium iodide (PI)‐positive (i.e., dead) cells (**Figure** **2**A). Staining with calcein‐AM (i.e., living cells) and PI confirmed a similar trend (Figure S3A, Supporting Information). We detected an increase in lipid peroxidation in YY2‐overexpressing HCT116, LoVo, and HT29 cells (Figure 2B), as well as shrinkage of mitochondria, a hallmark of ferroptosis caused by disruption of the mitochondrial membrane (Figure 2C). We treated YY2‐overexpressing HCT116 cells with ferrostatin‐1, an ROS scavenger that could inhibit ferroptosis, and found that viability of YY2‐overexpressing HCT116 cells could be restored in a ferrostatin‐1 dose‐dependent manner (Figure 2D), and was accompanied by fewer PI‐positive cells (Figure 2E). Addition of erastin, a ferroptosis inducer, increased the level of lipid peroxidation in HCT116 cells, but knocking out YY2 cancelled this effect (Figure S3B, Supporting Information). Concomitantly, HCT116 cells manifested significantly lower viability compared to HCT116^YY2null^ cells at every erastin concentration tested (Figure 2F). Furthermore, YY2‐silencing blocked the ability of erastin to induce cell death (Figure 2G; Figure S3C, Supporting Information). Immunofluorescence staining showed that knocking out YY2 abrogated the increase in PI‐positive cells caused by erastin and augmented the number of calcein‐AM‐positive cells (Figure S3D, Supporting Information). In contrast, YY2 overexpression restored the number of PI‐positive cells decreased by ferrostatin‐1 (Figure S3E, Supporting Information).

We next analyzed the level of lipid ROS and mitochondrial shrinkage in the xenograft formed by YY2‐overexpressing HCT116 cells. Staining with 4‐HNE showed that lipid peroxidation increased in tumors formed by YY2‐overexpressing HCT116 cells and decreased in those formed by HCT116^YY2null^ cells (Figure 2H). Transmission electron microscopy images showed that mitochondrial shrinkage was enhanced in xenografted tumors formed by YY2‐overexpressing HCT116 cells (Figure 2I). Taken together, our in vitro and in vivo results indicated that YY2 could induce ferroptosis, and that ferroptosis was critical for the tumor suppressive effect of YY2.

### 2.3 YY2 Lowers Cellular Cysteine and GSH Levels by Regulating SLC7A11

**Figure 3:**
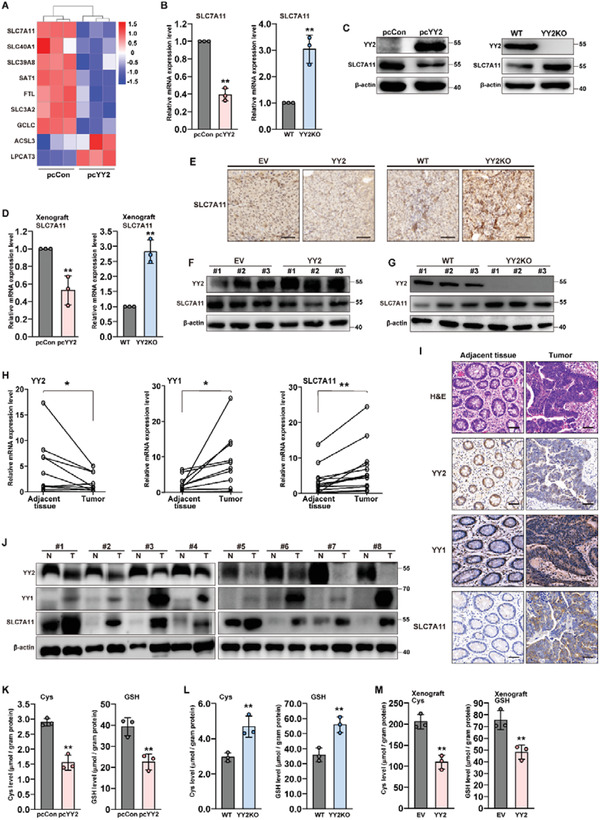
YY2 suppresses cellular cysteine and GSH levels by regulating SLC7A11. A) Heatmap showing differently expressed genes related to ferroptosis that are significantly enriched (*p* < 0.05). Values are scaled as indicated (1.5 to −1.5; n = 3). SLC7A11 B) mRNA and C) protein expression levels in HCT116 cells overexpressing YY2 and HCT116^YY2null^ cells, as determined using qRT‐PCR and western blotting, respectively. D) SLC7A11 mRNA expression level in xenografted tumors formed by HCT116 cells overexpressing YY2 (left) and HCT116^YY2null^ cells (right), as determined using qRT‐PCR. E) Immunohistochemical staining images against SLC7A11 in the tissue section of xenografted tumors formed by HCT116 cells overexpressing YY2 (left) and HCT116^YY2null^ cells (right). Scale bars, 50 *μ*m. YY2 and SLC7A11 protein expression levels in the xenografted tumors formed by F) HCT116 cells overexpressing YY2 and G) HCT116^YY2null^ cells, as determined by western blotting. H) YY2, YY1 and SLC7A11 mRNA expression levels in clinical human colon cancer and corresponding normal adjacent tissues (*n =* 13), as analyzed using qRT‐PCR. *p* values were calculated using two‐tailed paired Student's *t*‐test. YY2, YY1, and SLC7A11 protein expression levels in the clinical colon cancer tissue and corresponding normal adjacent tissue, as analyzed using I) immunohistochemistry staining and J) western blotting. Scale bars, 50 *μ*m. Cysteine (left) and GSH (right) levels in K) HCT116 cells overexpressing YY2 and L) HCT116^YY2null^ cells. M) Cysteine (left) and GSH (right) levels in the xenografted tumors formed by HCT116 cells overexpressing YY2 cells. Wild‐type HCT116 cells, cells transfected with pcCon or infected with empty lentivirus (EV) were used as controls. *β*‐actin was used for qRT‐PCR normalization and as western blotting loading control. Quantification data are shown as mean ± SD (*n =* 3, unless further indicated). *p* values were calculated using two‐tailed unpaired Student's *t‐*test. One‐way ANOVA analyses were performed when more than two groups were compared. pcCon: pcDNA3.1(+); YY2KO: HCT116^YY2null^ cells; ^**^ *p* < 0.01.

To elucidate the molecular mechanism of YY2‐induced ferroptosis, we used RNA‐Seq data and identified the fold‐change of differentially expressed genes related to ferroptosis in YY2‐overexpressing HCT116 cells (**Figure** **3**A). We confirmed their expression level in YY2‐overexpressing HCT116 cells using quantitative reverse‐transcription PCR (qRT‐PCR) (Figure S4A, Supporting Information). Both RNA‐Seq and qRT‐PCR pointed to SLC7A11 as the gene displaying the most significant change. The mRNA and protein levels of SLC7A11 were significantly downregulated in YY2‐overexpressing HCT116 cells, but robustly upregulated in HCT116^YY2null^ cells (Figure 3B,C) and YY2‐knocked down HCT116 cells (Figure S4B,C, Supporting Information).

Next, we examined the expression of SLC7A11 in xenografted tumors formed by YY2‐overexpressing HCT116 cells and HCT116^YY2null^ cells. qRT‐PCR showed that the SLC7A11 mRNA level was significantly downregulated in tumors formed by YY2‐overexpressing HCT116 cells, but upregulated in those formed by HCT116^YY2null^ cells (Figure 3D). Immunohistochemistry staining and western blotting further showed decreased SLC7A11 protein levels in tumors originated from YY2‐overexpressing HCT116 cells, as well as increased levels in tumors originated from HCT116^YY2null^ cells (Figure 3E–G).

Next, we analyzed the link between YY2 and SLC7A11 in clinical colorectal cancer tissues. YY2 mRNA was relatively low in tumor lesions compared to corresponding adjacent tissues; whereas YY1 and SLC7A11 mRNA was upregulated in tumor lesions (Figure 3H). This inverse correlation was confirmed by analyzing the protein levels of YY2, YY1, and SLC7A11 via immunohistochemistry and western blotting (Figure 3I,J).

As SLC7A11 is a transporter crucial for uptaking extracellular cystine, which is reduced to cysteine in the cells, we examined whether YY2 regulated the levels of intracellular cysteine and GSH. YY2 overexpression significantly reduced cysteine and GSH levels (Figure 3K), while its silencing significantly increased them (Figure 3L; Figure S4D, Supporting Information). Similarly, cellular cysteine and GSH levels were suppressed in xenografted tumors originated from YY2‐overexpressing HCT116 cells, but enhanced in those originated from HCT116^YY2null^ cells (Figure 3M; Figure S4E, Supporting Information).

In summary, these in vitro and in vivo results clearly showed that YY2 negatively regulated the expression of SLC7A11, thereby reducing intracellular cysteine and GSH levels.

### 2.4 SLC7A11 is Crucial for YY2‐Induced Ferroptosis and Tumor Suppression

**Figure 4:**
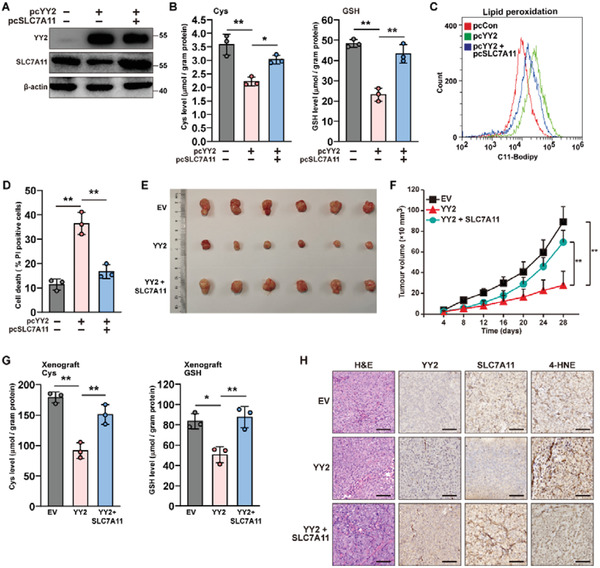
YY2 induces ferroptosis and tumor suppression by suppressing SLC7A11. A) YY2 and SLC7A11 protein levels in HCT116 cells overexpressing both YY2 and SLC7A11, as examined using western blotting. B) Cysteine (left) and GSH (right) levels in HCT116 cells overexpressing both YY2 and SLC7A11. C) Lipid peroxidation level in HCT116 cells overexpressing both YY2 and SLC7A11, as assessed by C11‐BODIPY staining and flow cytometry. D) Cell death percentage of HCT116 cells overexpressing both YY2 and SLC7A11, as measured by PI staining and flow cytometry. E,F) Tumorigenesis potential of HCT116 cells overexpressing both YY2 and SLC7A11 were examined in vivo by subcutaneous injection into Balb/c‐nu/nu mice (*n =* 6). Morphological images of the tumors generated at 28 days after injection (E) and volume of the tumors formed at indicated time points (F) are shown. G) Cysteine (left) and GSH (right) levels in the xenografted tumors formed by HCT116 cells overexpressing both YY2 and SLC7A11. H) Immunohistochemical staining images against YY2 and 4‐HNE in the tissue section of xenografted tumors formed by HCT116 cells overexpressing both YY2 and SLC7A11. Scale bars, 50 *μ*m. Cells transfected with pcCon or infected with empty lentivirus (EV) were used as controls. *β*‐actin was used as western blotting loading control. Quantification data are shown as mean ± SD (*n =* 3, unless further indicated). *p* values were calculated using two‐tailed unpaired Student's *t*‐test. One‐way ANOVA analyses were performed when more than two groups were compared. pcCon: pcDNA3.1(+); ^*^ *p* < 0.05; ^**^ *p* < 0.01.

To examine whether YY2 regulation of SLC7A11 was crucial for YY2‐induced tumor ferroptosis, we constructed a vector overexpressing SLC7A11 and two shRNA expression vectors targeting different sites on SLC7A11 (Figure S5A–C, Supporting Information). As the two shRNA expression vectors exerted similar suppressive effect, we chose shSLC7A11‐1 for further experiments. Overexpression of SLC7A11 restored SLC7A11 expression in addition to the cellular cysteine and GSH levels previously lowered by YY2 overexpression (**Figure** **4**A,B). At the same time, SLC7A11 knocking down prevented the increase in cysteine and GSH levels induced by YY2 knockout or knockdown (Figure S5D–G, Supporting Information).

Examination of typical ferroptosis characteristics revealed that SLC7A11 significantly suppressed lipid peroxidation (Figure 4C), and prevented cell death caused by YY2 overexpression (Figure 4D). Together, these results indicated that the regulatory action of YY2 on SLC7A11 was essential for inducing tumor ferroptosis, most likely via suppression of cellular cysteine and GSH levels.

Next, to determine whether SLC7A11 was essential for the YY2 tumor suppressive effect, we performed xenograft experiments using a stable HCT116 cell line overexpressing both YY2 and SLC7A11 (Figure S5H, Supporting Information). SLC7A11 overexpression abrogated the tumor‐suppressive effect of YY2 overexpression (Figure 4E,F). Concomitantly, SLC7A11 overexpression restored the cellular cysteine and GSH levels decreased by YY2 in xenografted tumors (Figure 4G). Immunohistochemistry staining revealed that while YY2 overexpression induced lipid peroxidation in xenograft tumor lesions, SLC7A11 overexpression suppressed it (Figure 4H). Hence, our results demonstrated that downregulation of SLC7A11 was essential for YY2 to induce ferroptosis and, consequently, for exerting a tumor‐suppressive effect.

### 2.5 YY2 Regulates SLC7A11 in a p53‐Independent Manner

**Figure 5:**
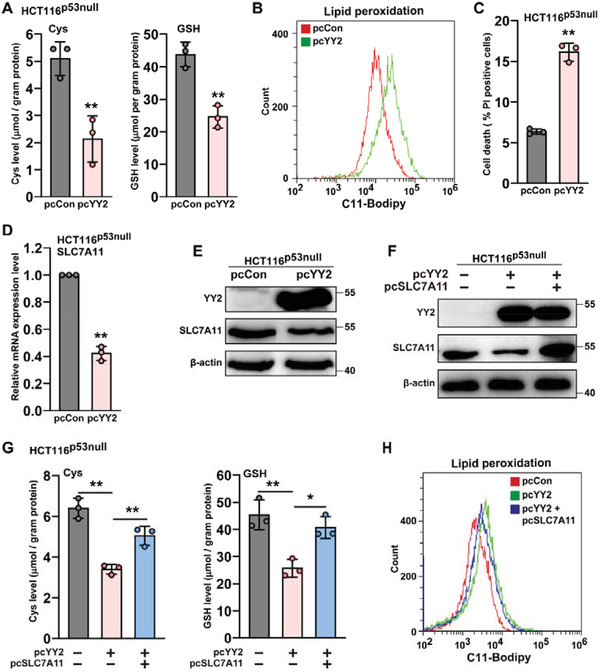
YY2 regulates ferroptosis in a p53‐independent manner. A) Cysteine (left) and GSH (right) levels in HCT116^p53null^ cells overexpressing YY2. B) Lipid peroxidation level in HCT116^p53null^ cells overexpressing YY2, as assessed by C11‐BODIPY staining and flow cytometry. C) Cell death percentage of HCT116^p53null^ cells overexpressing YY2, as assessed by PI staining and flow cytometry. SLC7A11 D) mRNA and E) protein expression levels in HCT116^p53null^ cells overexpressing YY2, as determined using qRT‐PCR and western blotting, respectively. F) YY2 and SLC7A11 protein expression levels in HCT116^p53null^ cells overexpressing both YY2 and SLC7A11, as examined using western blotting. G) Cysteine (left) and GSH (right) levels in HCT116^p53null^ cells overexpressing both YY2 and SLC7A11. H) Lipid peroxidation level in HCT116^p53null^ cells overexpressing both YY2 and SLC7A11, as assessed by C11‐BODIPY staining and flow cytometry. Cells transfected with pcCon were used as controls. *β*‐actin was used for qRT‐PCR normalization and as western blotting loading control. Quantification data are shown as mean ± SD (*n =* 3). *p* values were calculated using two‐tailed unpaired Student's *t*‐test. One‐way ANOVA analyses were performed when more than two groups were compared. pcDNA: pcDNA3.1(+); ^*^ *p* < 0.05; ^**^ *p* < 0.01.

Previous study demonstrated that p53, whose transcription could be enhanced by YY2,^[^ ^32^ ^]^ could induce ferroptosis by binding directly to the SLC7A11 promoter and blocking its transcription.^[^ ^33^ ^]^ To determine whether YY2 regulation of SLC7A11 occurred in a p53‐dependent manner, we overexpressed YY2 in HCT116^p53null^ cells (Figure S6A,B, Supporting Information). YY2 overexpression reduced cellular cysteine and GSH levels (**Figure** **5**A) and enhanced lipid peroxidation (Figure 5B) as well as cell death (Figure 5C) even in the absence of p53.

Furthermore, YY2 overexpression in HCT116^p53null^ cells lowered SLC7A11 mRNA and protein levels (Figure 5D,E), while knocking down YY2 in HCT116^p53null^ cells promoted them (Figure S6C,D, Supporting Information), suggesting that YY2 regulation of SLC7A11 could occur independently of p53. Overexpression of SLC7A11 in HCT116^p53null^ cells restored cellular cysteine and GSH levels decreased by YY2 (Figure 5F,G), and prevented lipid peroxidation (Figure 5H). In contrast, SLC7A11 silencing abrogated the increase in cellular cysteine and GSH caused by YY2 silencing (Figure S6E,F, Supporting Information). Hence, these results clearly showed that YY2 regulated SLC7A11 expression and, consequently, ferroptosis in a p53‐independent manner.

### 2.6 YY2/SLC7A11 Axis Regulates Tumorigenesis Independently of p53

**Figure 6:**
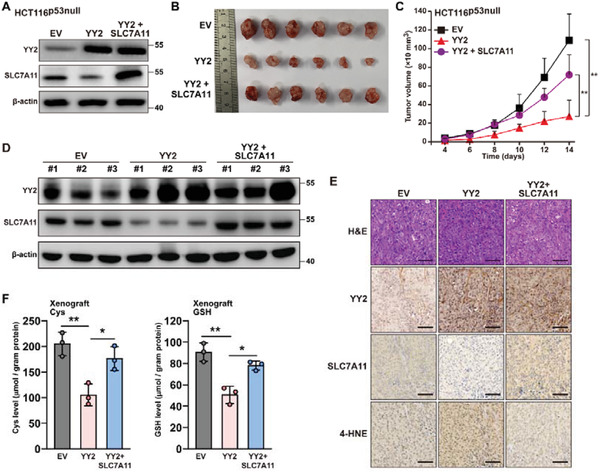
YY2 negatively regulates tumorigenesis by suppressing SLC7A11 in a p53‐independent manner. A) YY2 and SLC7A11 protein expression levels in HCT116^p53null^ cells infected with Lenti‐YY2 and Lenti‐SLC7A11, as determined using western blotting. B,C) Tumorigenesis potential of HCT116^p53null^ cells overexpressing YY2 and those overexpressing both YY2 and SLC7A11 were examined in vivo by subcutaneous injection into BALB/c‐nu/nu mice (*n  =*  6). Morphological images of the tumors generated at 14 days after injection (B) and volume of the tumors formed at indicated time points (C) are shown. D) YY2 and SLC7A11 protein expression levels in the xenografted tumors formed by HCT116^p53null^ cells, as determined by western blotting. E) Immunohistochemical staining images against YY2 and 4‐HNE in the tissue section of xenografted tumors formed by HCT116^p53null^ cells overexpressing both YY2 and SLC7A11. Scale bars, 50 *μ*m. F) Cysteine (left) and GSH (right) levels in xenografted tumors formed by HCT116^p53null^ cells overexpressing both YY2 and SLC7A11. Cells infected with empty lentivirus (EV) were used as controls. *β*‐actin was used as western blotting loading control. Quantification data are shown as mean ± SD (*n =* 3, unless further indicated). *p* values were calculated using two‐tailed unpaired Student's *t*‐test. One‐way ANOVA analyses were performed when more than two groups were compared. ^*^ *p* < 0.05; ^**^ *p* < 0.01.

To confirm the role of YY2 regulation on SLC7A11 expression in tumorigenesis, we performed xenograft experiments using HCT116^p53null^ cells stably overexpressing both YY2 and SLC7A11 (**Figure** **6**A). YY2 overexpression alone significantly reduced the size of xenografted tumors; however, SLC7A11 overexpression restored it (Figure 6B). Similarly, SLC7A11 abrogated the suppressive effect of YY2 overexpression on tumor growth rate (Figure 6C). Western blotting revealed a decrease in SLC7A11 expression in xenografted tumors formed by HCT116^p53null^ cells overexpressing YY2, while overexpression of SLC7A11 restored it (Figure 6D). These results were further confirmed by immunohistochemistry, which further demonstrated that lipid peroxidation increased in xenografted tumors formed by HCT116^p53null^ cells overexpressing YY2, but was blocked by overexpression of SLC7A11 (Figure 6E). Finally, we confirmed the restoration of cellular cysteine and GSH levels in xenografted tumors formed by HCT116^p53null^ cells overexpressing both YY2 and SLC7A11 (Figure 6F). Together, these findings indicated that the YY2/SLC7A11 pathway was crucial for the p53‐independent regulation of tumorigenesis through ferroptosis by regulating intracellular GSH, which was important for preventing lipid peroxidation.

### 2.7 Cancer‐Associated YY2 Mutations Abrogate YY2 Regulation on SLC7A11

**Figure 7:**
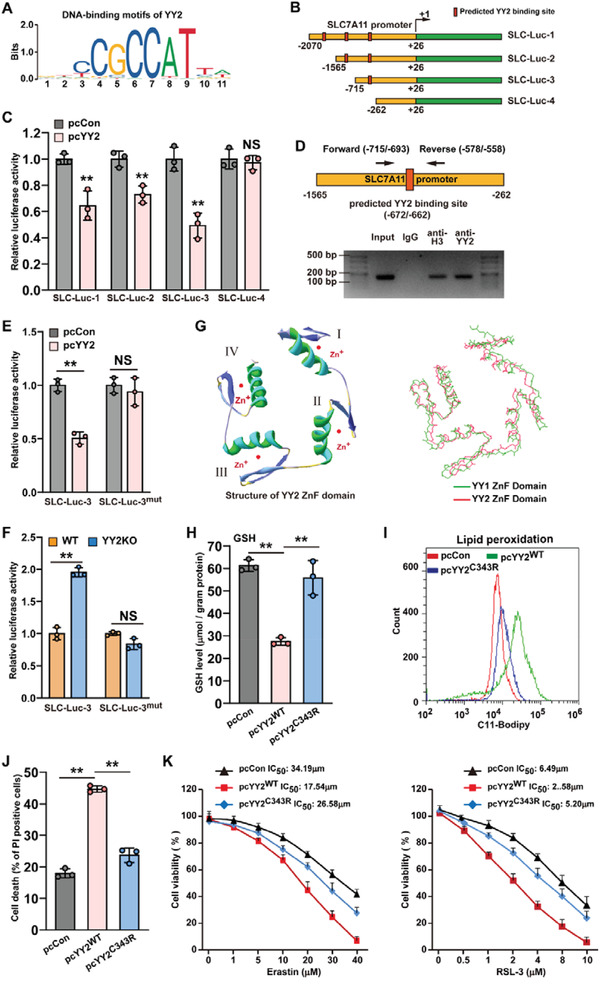
YY2 directly binds to SLC7A11 promoter and regulates its transcriptional activity. A) Schematic diagram of DNA‐binding motif of YY2 as predicted using JASPAR. B) Schematic diagram of SLC7A11 reporter vectors (SLC‐Lucs). C) Relative luciferase activities of SLC‐Luc‐1, SLC‐Luc‐2, SLC‐Luc‐3, and SLC‐Luc4 in HCT116 cells overexpressing YY2. D) Binding capacity of YY2 to the predicted region in the SLC7A11 promoter region, as determined using ChIP assay with an anti‐YY2 antibody followed by PCR. The predicted YY2‐binding site in the SLC7A11 promoter and the location of the primer pair used for PCR are shown. Relative activities of SLC‐Luc‐3 and SLC‐Luc^mut^ in E) HCT116 cells overexpressing YY2 and in F) HCT116^YY2null^ cells, as analyzed using dual luciferase reporter assay. G) Schematic diagram of YY2 protein domain structure. The ribbon structure of human YY2 zinc‐finger domains (amino acid residues 256 to 372) as predicted by AlphaFold Protein Structure Database (left panel), and comparison of human YY1 and YY2 zinc‐finger domains by Swiss‐PdbViewer (right panel) are shown. H) GSH levels in HCT116 cells overexpressing YY2^C343R^ mutant. I) Lipid peroxidation in HCT116 cells overexpressing YY2^C343R^ mutant, as assessed by C11‐BODIPY staining and flow cytometry. J) Cell death percentage of HCT116 cells overexpressing YY2^C343R^ mutant, as analyzed using PI staining and flow cytometry. K) Viability of HCT116 cells overexpressing YY2^C343R^ mutant after treatment with indicated dose of erastin or RSL‐3 for 48  h. Wild‐type HCT116, or cells transfected with pcCon or wild‐type YY2 expression vectors were used as controls. *β*‐actin was used as western blotting loading control. Quantification data are shown as mean ± SD (*n =* 3). *p* values were calculated using two‐tailed unpaired Student's *t*‐test. One‐way ANOVA analyses were performed when more than two groups were compared. pcCon: pcDNA3.1(+); YY2KO: HCT116^YY2null^ cells; ^**^ *p* < 0.01; NS: not significant.

We next explored the molecular mechanism underlying YY2 regulation of SLC7A11 expression. As mentioned above, YY2 regulated SLC7A11 mRNA expression in both HCT116 and HCT116^p53null^ cells, indicating that it controls SLC7A11 at the transcriptional stage. Prediction using JASPAR (http://jaspar.genereg.net)^[^ ^34^ ^]^ identified the binding motif of YY2 (**Figure** **7**A) and three possible YY2 binding sites at positions −1940 to −1930, −1293 to −1283, and −672 to −662 of SLC7A11 promoter, suggesting direct regulation of the SLC7A11 promoter by YY2. We then analyzed the effect of YY2 overexpression on the activity of firefly luciferase reporters coupled to different fragments of the SLC7A11 promoter (Figure 7B). YY2 overexpression significantly decreased the luciferase activity of SLC7A11‐Luc reporters coupled to the −2070 to +26 (SLC‐Luc‐1), −1565 to +26 (SLC‐Luc‐2), and −715 to +26 (SLC‐Luc‐3) regions, but not that coupled to the −262 to +26 regions (SLC‐Luc‐4) (Figure 7C), suggesting that the −715 to −261 region of the SLC7A11 promoter was essential for YY2‐mediated transcriptional suppression. The results of chromatin immunoprecipitation assay (ChIP) confirmed the binding of YY2 to the −715 to −558 region of the SLC7A11 promoter, further suggesting that the predicted YY2 binding site at −672 to −662 might be crucial for YY2 regulation on SLC7A11 promoter (Figure 7D).

Next, we constructed a firefly luciferase reporter vector coupled to the −715 to +26 region of the SLC7A11 promoter with mutations in the core of the predicted YY2 binding site (SLC7A11^mut^‐Luc, Figure S7A, Supporting Information). YY2 overexpression failed to suppress the activity of SLC7A11^mut^‐Luc; similarly, SLC7A11^mut^‐Luc activity displayed no difference between HCT116^YY2null^ and HCT116 cells (Figure 7E,F). Together, these results clearly suggested that YY2 could regulate SLC7A11 transcription via direct binding to the SLC7A11 promoter, most likely between positions −672 and −662.

To determine the critical functional domains of YY2 in regulating SLC7A11, we predicted the conformational structure of YY2 zinc‐finger domains, which are crucial for the activity of transcriptional factors, using AlphaFold Protein Structure Database (https://alphafold.ebi.ac.uk).^[^ ^35^ ^]^ The prediction result is shown in Figure 7G (left panel). A comparison between YY2 and YY1 zinc‐finger domains showed that the zinc‐finger domains of the two YY family members shared 86% homology in their amino acid sequence (Figure S7B, Supporting Information), and also possessed high similarity in their conformation (Figure 7G, right panel), indicating that similarly with YY1, the zinc‐finger domains might be crucial for the transcriptional activity of YY2. Using cancer genomics data set from cBioPortal for Cancer Genomics, we identified 149 somatic mutations on YY2, including 127 missense mutations, 20 nonsense mutations, and 2 in‐frame mutations.^[^ ^21^, ^36^ ^]^ Among the ten missense mutations with the highest functional impact scores as predicted using the Mutation Assessor from cBioPortal (http://mutationassessor.org/r2/), K263N, G317C, and C343R mutations are located at the first, third, and forth zinc‐finger domains of YY2, respectively, with C343R mutation as the most frequently found YY2 mutation in cancer patients. Our results showed that these three mutations significantly disrupted the suppressive effect of YY2 on SLC7A11 expression (Figure S7C, Supporting Information). Furthermore, unlike wild‐type YY2, YY2^C343R^ mutant almost failed to suppress cellular GSH level (Figure 7H), barely induced lipid peroxidation and cell death (Figure 7I,J), and scarcely suppressed colony formation potential (Figure S7D, Supporting Information). Moreover, as shown by the half‐maximal inhibitory concentration (IC_50_) values, unlike wild‐type YY2, YY2^C343R^ mutant failed to sensitize tumor cells to ferroptosis inducers (Figure 7K). Overall, these results indicate that mutations in the YY2 zinc‐finger domains as found in clinical tumor patients abrogated the YY2/SLC7A11 axis, and consequently, abrogated the effect of YY2 on tumor cell ferroptosis.

### 2.8 YY1 and YY2 Bind Competitively to the SLC7A11 Promoter and Antagonistically Regulate Tumor Cell Ferroptosis

**Figure 8:**
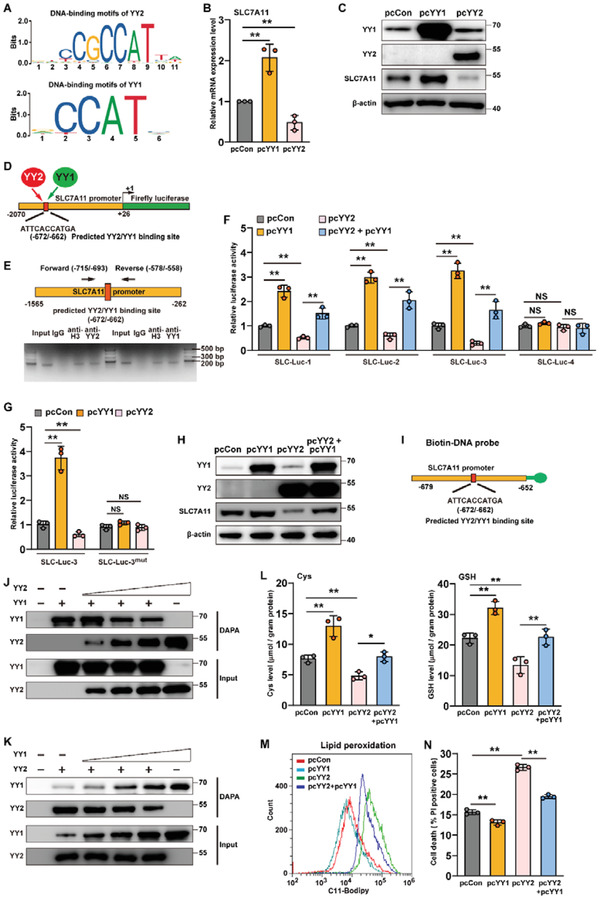
YY1 and YY2 oppositely regulates SC7A11 by competitive binding to SLC7A11 promoter. A) Schematic diagram of YY2 (upper panel) and YY1 (lower panel) DNA‐binding motifs, as predicted using JASPAR. SLC7A11 B) mRNA and C) protein expression levels in HCT116 cells overexpressing YY1 or YY2, as determined using qRT‐PCR and western blotting, respectively. D) Schematic diagram of common YY2 and YY1 binding site on SLC7A11 promoter, as predicted by JASPAR. E) Binding capacities of YY2 and YY1 to the predicted region in the SLC7A11 promoter, as determined using ChIP assay. Chromatin was immunoprecipitated separately using anti‐YY2 or anti‐YY1 antibodies from HCT116 cells overexpressing both YY1 and YY2, and subjected to PCR using the same primer pair. The predicted YY2 binding site in the SLC7A11 promoter and the location of the primer pair used for PCR are shown. F) Relative luciferase activities of SLC‐Luc‐1, SLC‐Luc‐2, SLC‐Luc‐3, and SLC‐Luc4 in HCT116 cells overexpressing YY1 and/or YY2, as examined using dual luciferase reporter assay. G) Relative activities of SLC‐Luc‐3 and SLC‐Luc^mut^ in HCT116 cells overexpressing YY1 and/or YY2, as examined using dual luciferase reporter assay. H) SLC7A11 protein expression level in HCT116 cells overexpressing YY1 and/or YY2, as determined using western blotting. I) Schematic diagram of biotinylated DNA probe for detecting the predicted YY2/YY1 binding site in SLC7A11 promoter. J,K) Competition of YY1 and YY2 binding on SLC7A11 promoter, as analyzed using DNA affinity precipitation assay (DAPA). L) Cysteine (left) and GSH (right) levels in HCT116 cells overexpressing YY1 and/or YY2. M) Lipid peroxidation level in HCT116 cells overexpressing YY1 and/or YY2, as assessed by C11‐BODIPY staining and flow cytometry. N) Cell death percentage of HCT116 cells overexpressing YY1 and/or YY2, as assessed by PI staining and flow cytometry. Cells transfected with pcCon were used as controls. *β*‐actin was used for qRT‐PCR normalization and as western blotting loading control. Quantification data are shown as mean ± SD (*n =* 3). *p* values were calculated using two‐tailed unpaired Student's *t*‐test. One‐way ANOVA analyses were performed when more than two groups were compared. pcCon: pcDNA3.1(+); ^*^ *p* < 0.05; ^**^ *p* < 0.01; NS: not significant.

As reported previously and as predicted using JASPAR, YY1 and YY2 have high homology and share common binding sites (**Figure** **8**A).^[^ ^11^, ^34^ ^]^ Hence, we next examined the effect of YY1 overexpression on SLC7A11 (Figure S8A, Supporting Information). In contrast to YY2, YY1 significantly promoted SLC7A11 expression (Figure 8B,C). Furthermore, we also predicted, using JASPAR,^[^ ^34^ ^]^ that YY1 could also bind to the −672 to −662 region of the SLC7A11 promoter (Figure 8D). To confirm this, we first performed ChIP assay by immunoprecipitating the chromatin from the same cell lysate overexpressing YY1 and YY2 using anti‐YY1 and anti‐YY2 antibodies separately, and amplified the precipitant using the same pair of primers flanking the predicted binding site. The results indicate that similar with YY2, YY1 could also bind to the −715 to −558 region of the SLC7A11 promoter (Figure 8E, lower panel). Contrary to YY2, YY1 overexpression increased the luciferase activity of SLC‐Luc‐1, SLC‐Luc‐2, and SLC‐Luc‐3; while YY2 overexpression abrogated the increased of the activity of these reporter vectors induced by YY1 overexpression (Figure 8F). It is also noteworthy that neither YY1 nor YY2 overexpression had any effect on SLC‐Luc‐4, indicating that the −715 to −261 region is crucial for both YY2 and YY1 regulation on SLC7A11 promoter. Moreover, YY1 failed to enhance the activity of SLC‐Luc^mut^ (Figure 8G), and its overexpression blocked the downregulation of SLC7A11 induced by YY2 overexpression (Figure 8H; Figure S8B, Supporting Information). These results suggested a competitive regulation by YY1 and YY2 of the same site on the SLC7A11 promoter.

To further explore the potential competitive transcriptional regulation of SLC7A11 by YY1 and YY2, we performed a DNA affinity precipitation assay (DAPA) with a biotin‐DNA probe targeting the −672 to −662 region of the SLC7A11 promoter, a constant amount of nuclear extract from YY1‐overexpressed HCT116^YY2null^ cells, and different amounts of nuclear extract from YY2‐overexpressed/YY1‐silenced wild‐type HCT116 cells, or vice versa (Figure 8I; Figure S8C, Supporting Information). The amount of YY1 bound to the biotin‐DNA probe decreased with an increase in lysate from YY2‐overexpressing cells; whereas that of YY2 increased (Figure 8J). Similarly, increasing amounts of lysate from YY1‐overexpressing cells reduced the amount of YY2 bound to the biotin‐DNA probe, while increasing that of YY1 (Figure 8K). YY1 overexpression also restored the cellular cysteine and GSH levels decreased by YY2 overexpression, as well as prevented lipid peroxidation and cell death (Figure 8L–N). These results clearly suggested that YY1 and YY2 acted antagonistically in regulating ferroptosis in tumor cells. Furthermore, these results show the possibility of the competitive binding and regulation of the same site on the SLC7A11 promoter by YY2 and YY1, thereby leading to opposite regulation of cystine transport.

To eliminate the competitive effect of YY1 and further confirm YY2 regulation on SLC7A11‐mediated ferroptosis and vice versa, we first constructed YY1‐knocked down HCT116 stable cell lines (Figure S9A, Supporting Information). As the suppressive effects of shYY1‐1 and shYY1‐2 were similar, we chose cells transfected with shYY1‐1 for further experiments. Similar to the results of overexpressing YY2 in HCT116 cells with YY1, YY2 overexpression still could significantly suppressed cell viability in YY1‐knocked down HCT116 cells (Figure S9B, Supporting Information). Furthermore, YY2 overexpression clearly suppressed SLC7A11 expression level and downregulated cellular cysteine, as well as GSH level in YY1‐knocked down cells (Figure S9C,D, Supporting Information). Concomitantly, lipid peroxidation also increased in YY1‐knocked down/YY2‐overexpressed HCT116 cells (Figure S9E, Supporting Information). These results further confirmed that YY2 could induce ferroptosis by suppressing SLC7A11 expression. Next, we examined the effect of YY1 overexpression in HCT116^YY2null^ cells, and confirmed that in the absence of YY2, YY1 overexpression could upregulate cellular cysteine as well as GSH level (Figure S10A, Supporting Information), downregulate lipid ROS level (Figure S10B, Supporting Information), and subsequently promoted cell viability (Figure S10C, Supporting Information). Thus, although due to their competitive binding and opposite regulation on SLC7A11 promoter, the effect of YY2 overexpression on ferroptosis in cells with both YY2 and YY1 might be an accumulative effect of the increase of YY2‐induced ferroptosis and the decrease of YY1‐suppressed ferroptosis, and vice versa, these results clearly showed that YY2 is a novel regulator of SLC7A11 that binds to its promoter and regulates its transcription.

**Figure 9:**
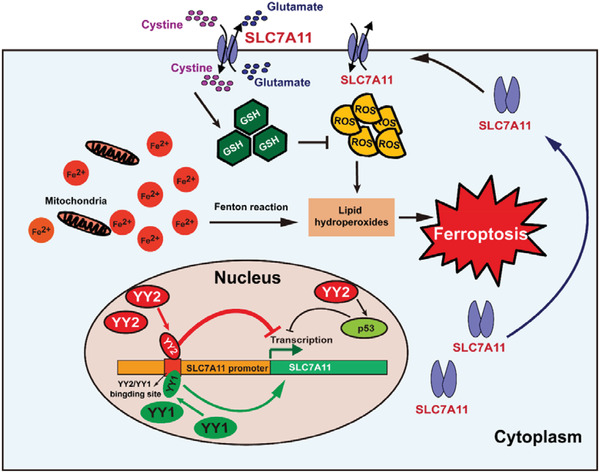
Schematic diagram showing the role of YY2 and YY1 in tumor cells ferroptosis through regulation of SLC7A11/GSH synthesis axis.

In summary, we describe a novel mechanism of ferroptosis in tumor cells based on the negative regulation exerted by YY2 on SLC7A11 transcription, and revealed a competitive, antagonistic regulation of YY1 and YY2 on the SLC7A11 promoter (**Figure** **9**).

## 3 Discussion

Ferroptosis is a non‐apoptotic form of cell death induced by redox stress, such as aberrant amino acid metabolism and ROS accumulation.^[^ ^3^, ^37^, ^38^ ^]^ Increased ROS causes lipid peroxidation which leads to irreversible damage of mitochondria and, consequently, ferroptotic cell death. In this study, we investigated a new regulatory mechanism of ferroptosis, whereby YY2 binds to and suppresses the activity of the SLC7A11 promoter. This in turn impairs cystine uptake and GSH synthesis, decreases antioxidant defenses in tumor cells, and suppresses tumorigenesis. YY2 could enhance activity of the *p53* promoter,^[^ ^32^ ^]^ while suppressing that of amino‐terminal enhancer of split, thereby inhibiting tumor metastasis.^[^ ^31^ ^]^ Furthermore, YY2 expression is aberrantly downregulated in various tumors, such as breast cancer and hepatocellular carcinoma, and its inactivation due to methylation at K279 enhances tumorigenesis.^[^ ^21^ ^]^ Nevertheless, there is currently little knowledge regarding the mechanism responsible for the tumor suppressive effect of YY2 or its role in regulating amino acid metabolic reprogramming in tumor cells. Our findings highlight an unprecedented relationship between YY2, amino acid metabolism, and ferroptosis, and show the critical role of YY2‐mediated regulation of ferroptosis on the tumor suppressive effect of YY2. Specifically, downregulation of the YY2/ferroptosis pathway represents an essential driver of tumorigenesis. Furthermore, mutations in YY2 zinc‐finger domains, which potentially could be found in clinical tumor patients, abrogated the YY2/SLC7A11 axis, leading to a decline of ferroptosis. Moreover, YY2 increases tumor cell sensitivity to ferroptosis inducers, suggesting the potential of using YY2 as a novel anti‐tumor therapeutic strategy. Given that ferroptosis also plays a crucial role in cardiovascular disease and neurological disorders,^[^ ^2^, ^39^, ^40^, ^41^ ^]^ our results indicate the possibility that the YY2/ferroptosis axis might also regulate other biological processes and pathological conditions.

Tumor cells are frequently exposed to elevated redox stress, and thus require abundant reducing force to maintain proliferation and tumorigenesis. This makes them more sensitive and prone to disruption of redox homeostasis by ferroptosis inducers. SLC7A11‐mediated cystine uptake is the rate‐limiting step of glutathione synthesis, which is critical for maintaining cellular redox homeostasis, eliminating lipid peroxidation, and thereby suppressing ferroptosis.^[^ ^1^, ^2^, ^5^, ^7^, ^37^, ^42^ ^]^ Upregulation of SLC7A11 has been identified in lung, breast, and colon cancers, where it promotes antioxidant defenses, favors tumorigenesis, and exhibits resistance against chemotherapy.^[^ ^5^, ^43^, ^44^ ^]^ This makes SLC7A11 an attractive target for anti‐tumor therapy. Indeed, ferroptosis inducers, which suppress SLC7A11 expression, have been shown to improve the efficacy of chemotherapy and radiotherapy by increasing the sensitivity of tumor cells to ROS.^[^ ^4^, ^44^, ^45^, ^46^ ^]^ Given that YY2 could suppress SLC7A11 expression, while further pre‐clinical and clinical investigations are needed, our study highlights the potential of combinatorial therapy using YY2 and chemotherapy or radiotherapy for treating tumors. Furthermore, as YY2 suppression on SLC7A11 expression could occur in a p53‐independent manner, our findings also suggest that targeting YY2/SLC7A11 axis, either alone or in combination with other therapies, is also promising for patients with p53 deletion and/or inactivation, which represents more than 50% of tumor patients.^[^ ^47^, ^48^ ^]^ Notably, as also being reflected by the enrichments in GO and KEGG, YY2 also affects the expression level of other factors related to ferroptosis, such as glutamate transporter spermidine/spermine N^1^‐acetyltransferase 1 (SAT1) and glutamate‐cysteine ligase catalytic subunit (GCLC) which are also involved in GSH biosynthesis, or solute carrier family 40 member 1 (SLC40A1), solute carrier family 39 member 8 (SLC39A8), and ferritin light chain (FTL), which are involved in iron metabolism;^[^ ^49^, ^50^, ^51^, ^52^, ^53^ ^]^ however, to lower extents to its regulation on SLC7A11. These facts suggest that while YY2 regulation on these factors might also affect ferroptosis, its regulation on SLC7A11 transcription plays a critical role for inducing ferroptosis and subsequently, suppressing tumorigenesis.

YY2 originated from retrotransposed YY1 mRNA, explaining their high DNA and amino acid homology. While having their own specific binding sites,^[^ ^54^ ^]^ they could also bind to the same sites on target promoters. However, while recent studies have provided further evidence of the tumor suppressive role of YY2 as opposed to the protooncogenic role of YY1, no studies have suggested that they could regulate common binding site in the same target gene antagonistically and exert opposite functions. We demonstrated that YY1 and YY2 might compete for regulation of the SLC7A11 promoter by binding to the same site, resulting in opposite effects on cysteine metabolism and ferroptosis, thus explaining their opposite regulation on tumorigenesis. Our findings indicate that YY1 and YY2 might antagonistically regulate the same target gene and ensuing phenotypes through competitive binding to the same site in the gene's promoter. Given that YY1 is upregulated while YY2 is downregulated in tumor cells, our results indicate that both an upregulation of YY1, which consequently impaired YY2 binding to the SLC7A11 promoter, and a downregulation of YY2 in tumor cells might enhance SLC7A11 expression and antioxidant defenses, and favor tumorigenesis. However, the fold‐change of YY1 and YY2 expression levels in tumor cells compared to normal cells might greatly varied among patients. Together with the affinities of YY1 and YY2 to SLC7A11 promoter, which also need to be elucidated, the change in YY1 and YY2 levels is crucial for the overall outcome of the ferroptosis level in tumor. Nevertheless, our findings characterize a novel mechanism employed by the YY family to maintain tumor cell redox homeostasis and promote tumorigenesis. Furthermore, as YY2 shares a common binding site with YY1, which is predicted to regulate around 7% of mammalian genes,^[^ ^55^ ^]^ the balance between YY1 and YY2 levels might also affect the expression of other target genes, and thus might be crucial for the regulation of other biological and pathological functions including tumorigenesis.

Zinc‐finger domains are critical for transcription factors for exerting their functions in regulating target genes transcription. While clinical data regarding YY2 are still limited, C343R, G317C, and K263N mutations in zinc‐finger domains of YY2 as potentially found in cancer patients cancelled the YY2 regulatory effect on SLC7A11 expression. It is noteworthy that the expression levels of YY2^C343R^ and YY2^G317C^ mutants were significantly lower than that of wild‐type YY2. Given that proteins with certain mutations, especially those which lead to misfolding and might cause damage to the cells, will be cleared by the cells, these facts suggest that tumor cells might not tolerate YY2 protein bringing the abovementioned mutations. Hence, these results indicate that certain mutations, including those in its zinc finger domains, abrogate YY2 expression, plausibly by altering its structure. It is also noteworthy that despite being expressed at a level similar to wild‐type YY2, the YY2^K263N^ mutant also failed to suppress SLC7A1, indicating that this mutation might disrupt the transcriptional regulator function of YY2. Thus, while further investigations are needed, these mutations might contribute to YY2 downregulation and loss‐of‐function in human tumor tissues, and subsequently, to clinical tumor development.

While our study showed the possibility of YY2 and YY1 to compete to bind to their common DNA binding site in the SLC7A11 promoter through biochemical approach in vitro, due to the complexity of transcriptional regulation, more advanced and sophisticated technologies are needed to clearly show their competition in living cells in the future. Furthermore, the mechanism underlying the opposite effect of YY2 and YY1 regulation on SLC7A11 promoter also remains to be elucidated. One possible mechanism for this distinct effect is their structural difference. Both YY2 and YY1 could exert transcriptional activator and suppressor functions, as they possess transcriptional activation domain and repression domain in their N and C termini, respectively. However, despite their high homology, previous studies have shown that there is slight difference between the zinc finger domains in YY2 and YY1.^[^ ^11^, ^54^ ^]^ Furthermore, unlike YY1, the N terminus of YY2 does not have the acidic‐rich domain and has a more stable structure, limiting the range of cofactors that could interact with it compared to YY1.^[^ ^56^ ^]^ Whether these structural differences underlie their different regulation on SLC7A11 remains to be elucidated.

In conclusion, we identified YY2 as a novel regulator of ferroptosis in tumor cells, which acted through the SLC7A11/GSH synthesis axis. Furthermore, we revealed the competitive regulation of YY family members on tumor cell redox homeostasis via competitive transcriptional regulation of SLC7A11. Our findings not only provide novel insights on the regulatory mechanism of ferroptosis, but also elucidate the molecular pathway underlying the tumor suppressive effect of YY2, as well as the antagonistic regulation of tumorigenic potential by the balance between YY1 and YY2.

## 4 Experimental Section

### Cell lines and Cell Cultures

Wild‐type HCT116, MHCC‐97H, MCF‐7, HT29, and 293T cell lines were purchased from the Cell Bank of Chinese Academy of Sciences (Shanghai, China), and cultured in Dulbecco's modified Eagle's medium (Gibco, Life Technologies, Grand Island, NY) supplemented with 10% fetal bovine serum (FBS; Biological Industries, Beit Haemek, Israel) and 1% penicillin‐streptomycin. LoVo cell lines were purchased from the Cell Bank of Chinese Academy of Sciences (Shanghai, China), and cultured in F12K Ham's Kaighn's Modification medium (Macgene, Beijing, China), supplemented with 10% FBS (Biological Industries) and 1% penicillin‐streptomycin. p53‐null HCT116 (HCT116^p53null^) cells were kindly provided by Dr. Bert Vogelstein at John Hopkins University School of Medicine, and maintained in McCoy's 5A medium (Gibco, Grand Islands, NY) with 10% FBS (Biological Industries) and 1% penicillin‐streptomycin. Cell lines were verified using short‐tandem repeat profiling method, and were tested periodically for mycoplasma contamination by using Mycoplasma Detection Kit‐QuickTest (Biotool, Houston, TX).

### Animal Experiments

For the in vivo tumor study, BALB/c‐nu/nu mice (male, body weight: 18–22 g, 6 weeks old) were purchased from the Chongqing Medical University (Chongqing, China). Animal studies were approved by the Institutional Ethics Committee of Chongqing Medical University, and carried out in the Chongqing Medical University. All animal experiments conformed to the approved Guidelines for the Care and Use of Laboratory Animals of Chongqing Medical University (Permit No. SYXK‐2018‐003). All efforts were made to minimize suffering.

For xenograft experiments, BALB/c‐nu/nu mice were randomly divided into 3 groups (*n =* 6), and each group was injected subcutaneously with 3 × 10^6^ indicated stable cell lines. Tumor size (*V*) was evaluated by a caliper every 2 days using the following equation: *V* = *a* × *b* ^2^/2, where *a* and *b* are the major and minor axes of the tumor, respectively. The investigator was blinded to the group allocation and during the assessment.

### Plasmids and Constructs

shRNA expression vectors targeting two different sites of YY2, YY1, and SLC7A11 were constructed as described previously.^[^ ^26^ ^]^ Target sites were designed using the algorithm previously reported, and the sequences were as follows: shYY2‐1: 5″‐GCA TCA ACA TCA ACA TCA A‐3″; shYY2‐2: 3″‐ACA TCA ACA TCA ACC CAG A‐3″; shYY1‐1: 5′‐GCA AGA AGA GTT ACC TCA G‐3′; shYY1‐2: 5′‐GTC CAG AAT ACT TAT AAT T‐3′; shSLC7A11‐1: 5″‐GGA ACA ACT ATA AAG AAA T‐5″; and shSLC7A11‐2: 3″‐GGT CAA ACG CAG AAC TTT A‐5″. YY2 and YY1 overexpression vectors (pcYY2 and pcYY1, respectively) were constructed as described previously.^[^ ^26^, ^32^ ^]^ For SLC7A11 overexpression vector, the coding region of human SLC7A11 were amplified using the Takara Ex Taq Kit (Takara Bio, Dalian, China) from human cDNA obtained by reverse‐transcribing total RNA extracted from HCT116 cells using the PrimeScript RT Reagent Kit with gDNA Eraser (Takara Bio). The amplicon was inserted into the *Kpn*I and *Eco*RI sites of pcDNA3.1(+) (Invitrogen Life Technologies, Carlsbad, CA).

For reporter vectors bringing different regions of SLC7A11 promoter (Refseq No. NC_000004.12; SLC7A11‐Luc‐1 with the −2070 to +26 region; SLC7A11‐Luc‐2 with the −1565 to +26 region, SLC7A11‐Luc‐3 with the −715 to +26 region, and SLC7A11‐Luc‐4 with the −262 to +26 region), the corresponding regions of the SLC7A11 promoter were cloned into the *Bgl*II and *Bln*I sites of the pGL4.13 vector (Promega, Madison, WI). Human genome DNA was extracted from HCT116 cells using Genomic DNA Kit (Tiangen Biotech, Beijing, China), and was used as template. Promoter regions were then amplified using Takara PrimeSTAR Max DNA Polymerase (Takara Bio). SLC7A11 luciferase reporter vector with mutated YY2 binding site (SLC7A11‐Luc^mut^), as well as cancer‐associated mutant YY2 overexpression vectors (K263N, G317C, and C343R), were constructed using Site‐directed Mutagenesis Kit (Beyotime Biotechnology, Shanghai, China).

For construction of lentivirus vectors overexpressing YY2 and SLC7A11 (pLenti‐YY2 and pLenti‐SLC7A11, respectively), the coding region of YY2 and SLC7A11 were amplified from human cDNA obtained as described above using Takara PrimeSTAR Max DNA Polymerase (Takara Bio). The coding region of YY2 was inserted into the *Eco*RI and *Bam*HI sites, while that of SLC7A11 was inserted into the *Eco*RI and *Sma*I sites of lentivirus vector pCDH‐CMV‐MCS‐EF1‐Puro.

### Clinical Human Colon Carcinoma Specimen

Human colon carcinoma specimens were obtained from colon carcinoma patients undergoing surgery at Chongqing University Cancer Hospital (Chongqing, China), and stored in Biological Specimen Bank of Chongqing University Cancer Hospital. Patients did not receive chemotherapy, radiotherapy, or other adjuvant therapies prior to the surgery. The specimens were snap‐frozen in liquid nitrogen. Prior patient's written informed consents were obtained. The experiments were approved by the Institutional Research Ethics Committee of Chongqing University Cancer Hospital (Permit No. CZLS2021251‐A), and conducted in accordance with Declaration of Helsinki.

### RNA Sequencing and Data Analysis

Plasmids (pcCon and pcYY2) were transfected into HCT116 cells. RNA extraction and RNA‐Seq analysis using Illumina HiSeq 2500 (Illumina; three repetitive for each group), were performed by Shanghai Bio Technology Corporation (Shanghai, China). Sequencing raw reads were pre‐processed by filtering out rRNA reads, sequencing adapters, short‐fragment reads and other low‐quality reads. Tophat v2.1.0 was used to map the cleaned reads to human reference genome ensemble GRCh38 (hg38) with two mismatches. After genome mapping, Cufflinks v2.1.1 was run with a reference annotation to generate FPKM values for known gene models. Differentially expressed genes were identified using Cuffdiff. The *p*‐value significance threshold in multiple tests was set by the false discovery rate (FDR). The fold‐changes were also estimated according to the FPKM in each sample. The differentially expressed genes were selected using the following filter criteria: FDR ≤ 0.05.

### Cell Experiments and Cell Transfection

For gene‐silencing experiments, cells were seeded in 6‐well plate and transfected with 2 *μ*g of indicated vectors. 24 h after transfection, transfected cells were selected using 1 µg mL^−1^ puromycin for 36 h. For gene overexpression experiments, cells were seeded in 6 well‐plates, transfected with 2 *μ*g of indicated vectors, and collected 24 h after transfection for further experiments. For double silencing experiments, cells were transfected with 1 *μ*g of each indicated vectors, and subjected to puromycin selection to eliminate untransfected cells. YY2‐knocked out HCT116 (HCT116^YY2null^) cells were established using CRISPR/Cas9 method. Briefly, cells were transfected with vectors targeting YY2 (HCP301990‐CG04‐3‐10‐a, target site: 5″‐GAT GGC AAT TGG ATC TAC GG‐3″; HCP301990‐CG04‐3‐10‐b, target site: 3″‐TAG CCC GTG TTC GTG AAG AG‐5″; HCP301990‐CG04‐3‐10‐c, target site: 3″‐TCC GTC GGA ATG TCC TCC AT‐5″; Gene Copoiea, Rockville, MD). 24 h later, neomycin selection (600 ng mL^−1^) was performed for 10 days to eliminate untransfected cells. Cell line was then established from a single clone. The corresponding genome DNA was subjected to sequencing, and deletion of nucleotides located in +95 to +151 region (56 bp) of YY2 coding sequence was confirmed. All transfections were performed using Lipofectamine 2000 (Invitrogen Life Technologies) according to the manufacturer's instruction.

### Lentiviruses Packaging and Infection

Lentiviruses were generated by co‐transfecting 293T cells with 8 *μ*g pLenti‐YY2 or pLenti‐SLC7A11 vectors, 6 *μ*g pCMVΔR, and 2 *μ*g pCMV‐VSVG using Lipofectamine 2000 in a 10 cm dish. Medium was changed at the following day and lentivirus‐containing supernatant was harvested and filtered with a 0.45‐µm filter after 48 h. For infection, HCT116 cells were cultured in a 6‐well plate. 24 h later, the medium was changed with 1 mL fresh culture medium and 1 mL virus supernatant. Infected cells were then selected using 1 *μ*g mL^−1^ puromycin for 7 days. The expression of the gene of interest was confirmed using western blotting.

### DNA Affinity Precipitation Assay

Biotin was conjugated to the 3′‐end of the oligonucleotide representing the sense strand of −679 to −652 bp of the SLC7A11 promoter (5″‐TCT GGA GTC ATG GTG AAT TTT GTA TTA G‐3″), which contained the predicted YY2 and YY1 common binding site. Then the biotinylated sense strand was annealed with the oligonucleotide representing the corresponding antisense strand to form the biotin‐conjugated double‐stranded DNA probe. Next, 40 *μ*L of streptavidin magnetic beads (Macklin, Shanghai, China) was added to 400 pmol of the biotin‐conjugated double‐stranded oligonucleotide. The mixture was then incubated at 4 °C for 2 h, then the DNA‐streptavidin magnetic beads complex was washed three times with lysis buffer. For extraction of cell lysates, HCT116^YY2null^ cells overexpressing YY1 and YY2‐overexpressed/YY1‐silenced HCT116 cells were prepared as indicated above, collected, and washed twice with PBS. Nuclear protein was extracted using the Nuclear and Cytoplasmic Protein Extraction Kit (KeyGene, Nanjing, China). Briefly, cells were lysed in ice‐cold lysis buffer for 30 min and centrifuged for 10 min (3000 rpm, 4 ℃) to remove the supernatant which contained cytoplasmic protein. Nuclei were then lysed using lysis buffer, and incubated on ice for 1 h in lysis buffer. Nuclear proteins obtained by centrifugation for 30 min (12 000 rpm, 4 ℃) were incubated with DNA‐streptavidin magnetic beads complex for 2 h at 4 °C. The protein‐DNA‐streptavidin magnetic beads complex was washed three times with PBS and loaded onto a polyacrylamide gel for SDS‐PAGE followed by western blotting using corresponding antibodies. For experiments with constant amount of nuclear extract from YY1‐overexpressed HCT116 cells and different amount of nuclear extract from YY2‐overexpressed cells, 60 *μ*g of nuclear extract from YY1‐overexpressed HCT116^YY2null^ cells and 50, 100, 150, or 300 *μ*g of nuclear extract from YY2‐overexpressed/YY1‐silenced HCT116 cells were used, and vice versa.

### Chromatin Immunoprecipitation Assay

Chromatin was immunoprecipitated using the ChIP Assay Kit (Beyotime Biotechnology) according to the manufacturer's instruction. Briefly, cells were lysed and chromatins were then immunoprecipitated using protein A+G Agarose/Salmon Sperm DNA and anti‐YY2 antibody, anti‐YY1 antibody, or normal mouse IgG, de‐crosslinked for 4 h at 65 °C, and treated with 0.5 m EDTA, 1 m Tris (pH 6.5), and 20 mg mL^−1^ proteinase K. Immunoprecipitated chromatin was then subjected to PCR by using PrimeSTAR Max (Takara Bio). Primer sequences for amplifying the SLC7A11 promoter region with the predicted YY2 and YY1binding site were: 5″‐CAC CTA GTG CTA ATG AGA ATC AG‐3″ (forward primer); and 5″‐CAC ACA ACT ATA AGC CTT CC‐3″ (reverse primer).

### Immunoprecipitation Assay

Cells were seeded in a 10 cm dish (4 × 10^6^ cells per dish) and transfected with 8 *μ*g pcYY1 and 8 *μ*g pcYY2. After 48 h, cells were harvested and lysed with 1 mL immunoprecipitation lysis buffer containing protease inhibitor and phosphatase inhibitor cocktail (complete cocktail, Roche Applied Science, Mannheim, Germany) followed by centrifugation at 12 000 rpm for 10 min at 4 °C. Supernatants were immunoprecipitated with anti‐YY2, anti‐YY1, or anti‐IgG protein A+G beads (Beyotime Biotechnology) as control for 4 h at 4 °C. Beads were washed three times with immunoprecipitation buffer at 4 °C and boiled at 100 °C for 5 min. Aliquots were subjected to immunoblotting analysis.

### Lipid Peroxidation Assay

To detect lipid peroxidation, cells were incubated with 5 *μ* m BODIPY 581/591 C11 (Invitrogen Life Technologies) in the dark for 30 min at 37 °C. Cells were harvested by trypsinization, washed twice with PBS, and re‐suspended in 300 *μ*L PBS. Fluorescence was analyzed using a flow cytometer (Beckman Coulter, Inc., California, USA) equipped with a 488 nm laser for excitation, and data were collected using the 530 nm band‐pass filter.

### GSH and Cysteine Assay

To measure the levels of GSH and cysteine in cultured cells, cells were transfected with indicated vectors as described above, harvested by trypsinization, and collected by centrifugation. The pellets were then dissolved in protein stripping buffer. To measure the levels of GSH and cysteine in xenografted tissues, the tissues were homogenized with protein stripping buffer. The levels of total GSH and cysteine were then measured using the Total GSH Assay (Beyotime Biotechnology) and Cysteine Assay (Solarbio, Beijing, China) kits. The values were normalized with total protein amount determined using the BCA Protein Assay Kit (Beyotime Biotechnology).

### Transmission Electron Microscopic Analysis

Cells and xenograft tissue samples were fixed with 2.5% glutaraldehyde, washed in 0.1 m phosphate buffer (pH 7.4), and post‐fixed with 1% osmium ∙ 0.1 m phosphate buffer. Samples were dehydrated in increasing concentrations of ethanol, infiltrated, and embedded in SPI‐Pon812 before being polymerized in a 60 °C oven for 48 h. Ultrathin sections were cut using a Leica Ultracut microtome and loaded on formvar and carbon‐coated copper grids. Grids were photographed using transmission electron microscope (HITACHI HT7700, Tokyo, Japan).

### RNA Extraction and Quantitative Reverse‐Transcription PCR

Total RNA was extracted using Trizol (Invitrogen Life Technologies) according to the manufacturer's instruction. Total RNA (1 *μ*g) was then reverse‐transcribed into cDNA using PrimeScript Reagent Kit with gDNA Eraser (Takara Bio). qRT‐PCR was performed using SYBR Premix ExTaq (Takara Bio). The sequences of the primers used are listed in Table S1, Supporting Information. *β*‐Actin was used to normalize sample amplification. The results are shown as relative to the expression level in the corresponding controls, which are assumed as 1.

### Western Blotting

Total cells were lysed with RIPA lysis buffer with protease inhibitor and phosphatase inhibitor cocktail (complete cocktail; Roche Applied Science). Equal amounts of the sample proteins were electrophoresed on sodium dodecyl sulphate polyacrylamide gel before being transferred to a polyvinylidene fluoride membrane with 0.45 *μ*m pores (Millipore, Billerica, MA). Membrane was then incubated with first antibodies followed by second antibodies. Antibodies used are listed in Table S2, Supporting Information, and immunoblotting with anti‐*β*‐actin antibody was conducted to ensure equal protein loading. Signals were measured using Super Signal West Femto Maximum Sensitivity Substrate detection system (Thermo Scientific, Waltham, MA). Images of uncropped blots are shown in Figure S11A–N, Supporting Information.

### Dual Luciferase Reporter Assay

Cells were seeded into 24‐well plates (8 × 10^4^ cells per well). 24 h later, cells were co‐transfected with indicated shRNA expression vectors or overexpression vectors, reporter vector, and *Renilla* luciferase expression vector (pRL‐SV40, Promega) as internal control. 48 h after co‐transfection, luciferase activities were measured with Dual Luciferase Assay System (Promega). Relative light units of firefly luciferase were normalized to the corresponding *Renilla* luciferase activities. The results are shown as relative to the expression level in the corresponding controls, which are assumed as 1.

### Cell Viability Assay

Cells were transfected with indicated vectors, and 24 h after transfection, puromycin selection was performed as described previously. Cells were then re‐seeded into 96‐well plates at the density of 5 × 10^3^ cells per well. Cell numbers were measured by colorimetric assays with 3‐(4,5‐dimethylthiazol‐2‐yl)‐5‐(3‐carboxymethoxyphenyl)‐2‐(4‐sulfophenyl)‐2H‐tetrazolium (MTS, Promega) in accordance with the manufacturer's instructions at indicated time points. Cell viabilities were obtained as the ratio of the cell numbers to those of mock (untransfected) cells at corresponding time point.

### Colony Formation Assay

Cells were transfected with indicated vectors and selected using puromycin as described above. Cells were then re‐seeded into 6‐well plates at a density of 300 cells per well, cultured for 8 days, and then fixed with 30% paraformaldehyde and stained with methylene blue. Quantification was then performed by counting the number of the colonies formed. Investigator was blinded during the assessment.

### Cell Death Assay

Cells were transfected with indicated vectors and selected with puromycin as described above. Cells were then re‐seeded in 6‐well plate (3 × 10^5^ cells per well). 24 h later, cell death quantification was performed by staining with PI (NeoBiosciences, Shanghai, China) followed by FACS analysis.

### Immunohistochemistry and Hematoxylin‐Eosin Staining

Fresh human colon cancer tissues, normal adjacent tissues, and xenografted tumors were fixed using 4% paraformaldehyde for overnight prior to being embedded in paraffin and sectioned at 4 µm thickness using a cryostat. Sections were then dewaxed using xylene, rehydrated, and subjected to immunohistochemical staining. Briefly, the tissue sections were incubated with primary antibodies for 1 h, following by incubation with corresponding secondary antibodies conjugated with horse‐radish peroxidase. Visualization was performed using a DAB Kit (DAKO, Beijing, China) under microscope. The nuclei were then counterstained with hematoxylin (Beyotime), followed by dehydration and coverslip mounting. The antibodies used are listed in Table S2, Supporting Information. Images were taken using Pannoramic Midi (3DHistech, Budapest, Hungary).

For hematoxylin‐eosin (H&E) staining, paraffin sections from human colon cancer tissues and normal adjacent tissues, as well as from mice subcutaneous tumors generated in xenograft experiment (4 *μ*m thickness) were fixed in 10% formalin and washed with 60% propylene glycerol. The samples were then stained with 0.5% H&E (Sangon Bio, Shanghai, China) for 3 min followed by dehydration and coverslip mounting. Images were taken using Pannoramic Midi.

### Calcein‐AM/PI Staining

Cells were transfected with indicated vectors, selected with puromycin as described above, and re‐seeded in 24‐well plate (1 × 10^5^ cells per well). 24 h later, the medium was removed and the cells were washed with PBS for 5 min at 37 °C. Cells were then incubated with medium containing 2 µm Calcein‐AM at 37 °C for 20 min. After being washed twice with PBS, cells were incubated with medium containing 4.5 µm PI at 37 °C for 5 min, and washed twice with PBS. Images were taken using fluorescence microscope (Leica, Heidelberg, Germany).

### Statistical Analysis

All quantification results were presented as mean ± S.D. (*n =* 3, unless further indicated). Statistical analysis was performed using two‐tailed unpaired Student's *t*‐test conducted using GraphPad Prism 9.0. When more than two groups were compared, one‐way ANOVA analyses were performed. A value of *p* < 0.05 was considered statistically significant.

## Conflict of Interest

The authors declare no conflict of interest.

## Author Contributions

Conceptualization: S.W., V.K.; methodology: V.K., S.W., Y.L., H.Z., and M.M.; investigation: Y.L., J.L., Z.L., and M.W.; visualization: V.K., S.W., and Y.L.; supervision: V.K., S.W.; writing—original draft: V.K., S.W., and Y.L.; writing—review and editing: V.K., S.W.

## Supporting information

Supporting Information

## Data Availability

The data that support the findings of this study are available from the corresponding author upon reasonable request.
