# Supplementary material for: Homeostasis Imbalance of YY2 and YY1 Promotes Tumor Growth by Manipulating Ferroptosis
Source: Adv Sci (Weinh). 2022 Mar 4;9(13):2104836. doi: 10.1002/advs.202104836 (PMC9069185; doi:10.1002/advs.202104836)
Supplement: Supplementary file 1 — Supporting Information [file ADVS-9-2104836-s001.pdf]

# Supplementary Materials for

## Homeostasis imbalance of YY2 and YY1 promotes tumor growth by manipulating ferroptosis

*Yanjun Li, Juan Li, Zhuolin Li, Mankun Wei, Hezhao Zhao, Makoto Miyagishi, Shourong Wu*, Vivi Kasim**

*E-mail: vivikasim@cqu.edu.cn (V.K.)
shourongwu@cqu.edu.cn (S.W.)

**This PDF file includes:**

**Figure S1**. The efficacy and specificity of *YY2* overexpression vector, shRNA expression vectors targeting *YY2*, and antibodies used.

**Figure S2**. YY2 suppresses tumor cells viability.

**Figure S3**. YY2 regulates tumor cells ferroptotic cell death.

**Figure S4**. *YY2*-silencing suppresses SLC7A11 and induces cellular cysteine and GSH levels.

**Figure S5**. SLC7A11 is crucial for YY2 suppression on cellular cysteine and GSH levels.

**Figure S6**. YY2 regulates SLC7A11/GSH synthesis axis in a p53-independent manner.

**Figure S7**. Mutation in zinc finger domains abolished YY2 regulatory effect on SLC7A11 expression.

**Figure S8**. YY2 and YY1 regulate SLC7A11 expression level.

**Figure S9**. YY2 regulates SLC7A11/GSH synthesis axis in *YY1*-knocked down cells.

**Figure S10**. YY1 regulates ferroptosis in HCT116$^{YY2null}$ cells.

**Figure S11**. Uncropped western blots with the indicated areas of selection in Figures 1, 3 to 6 and 8, Figures S1, S2, and S4 to S9.

**Table S1**. Primer pairs used for gene quantification by quantitative RT-PCR.

**Table S2**. Antibodies used for western blotting, immunohistochemical staining and ChIP assay.

**Figure S1.**

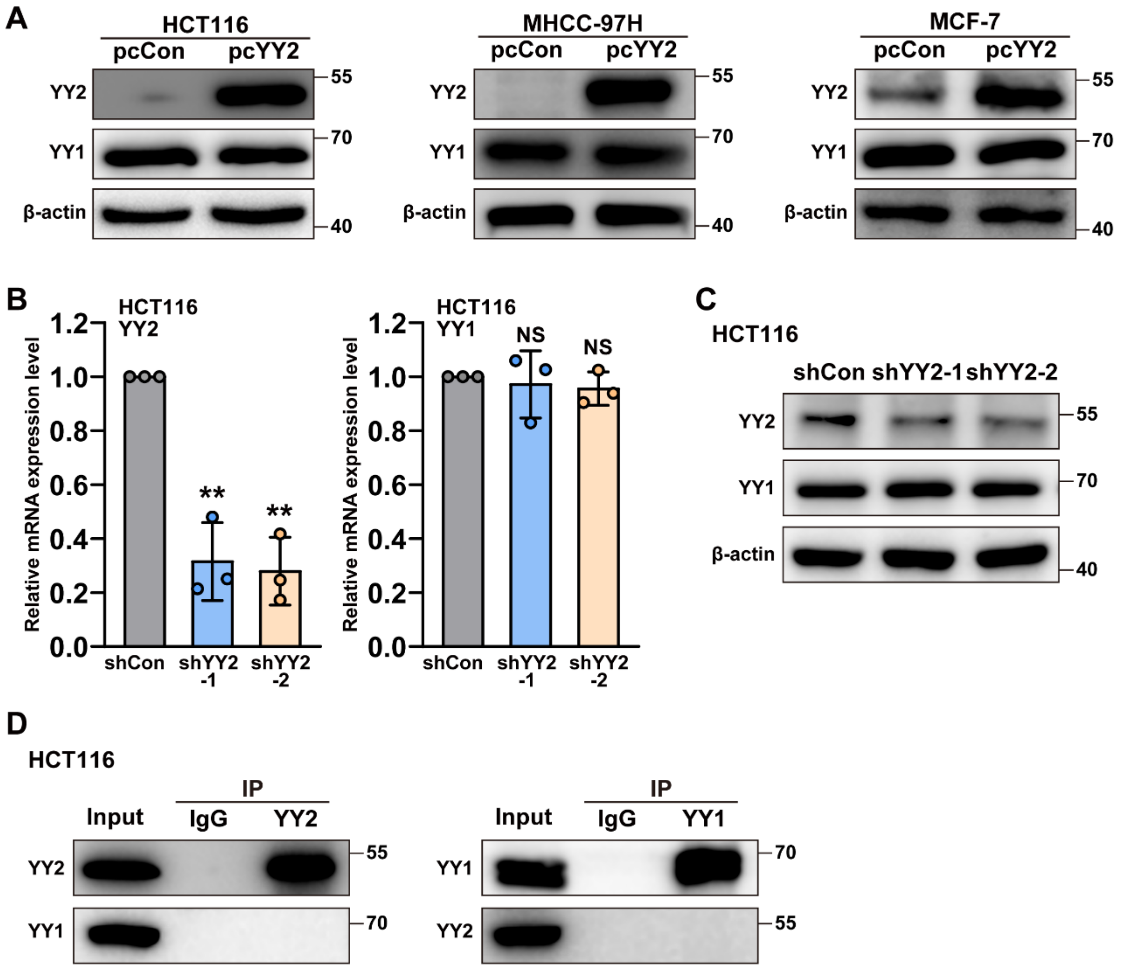


**Supplementary Fig. S1. The efficacy and specificity of *YY2* overexpression vector, shRNA expression vectors targeting *YY2*, and antibodies used. (A)** YY2 protein expression level in HCT116, MHCC-97H, and MCF-7 cells transfected with *YY2* overexpression vector, as determined using western blotting. **(B and C)** YY2 and YY1 mRNA (B) and protein (C) expression levels in HCT116 cells transfected with shRNAs targeting *YY2*, as determined using quantitative reverse-transcription PCR (qRT-PCR) and western blotting, respectively. **(D)** Specificity of the YY2 and YY1 antibodies used in this work, as determined using immunoprecipitation assay. Cells transfected with shCon or pcCon were used as controls. β-actin was used for qRT-PCR normalization and as western blotting loading control. Quantification data are shown as mean ± SD (n = 3). *P* values were calculated using two-tailed unpaired Student's t-test. pcCon: pcDNA3.1(+); $^{**}P < 0.01$.

## Figure S2.

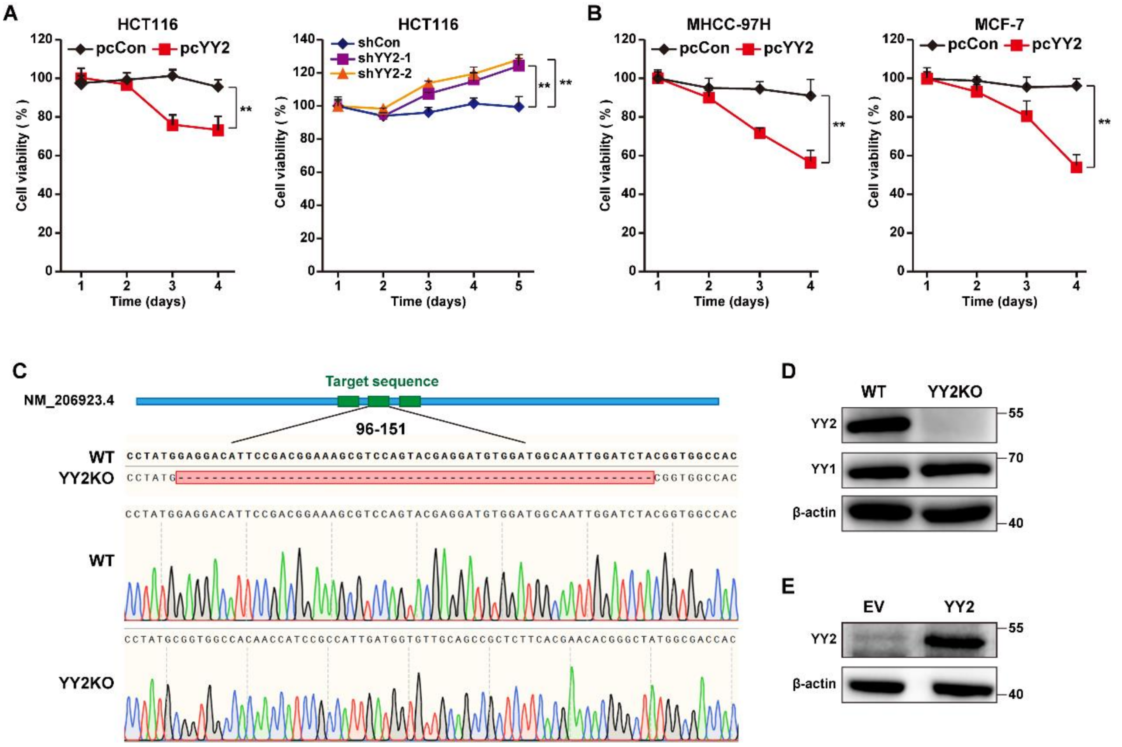


**Supplementary Fig. S2. YY2 suppresses tumor cells viability. (A)** Viabilities of *YY2*-overexpressed (left panel) and *YY2*-knocked down (right panel) HCT116 cells were measured at indicated time points. **(B)** Viabilities of MHCC-97H and MCF-7 cells overexpressing *YY2* were measured at indicated time points. **(C)** Establishment of *YY2*-knocked out HCT116 (HCT116$^{YY2null}$) cells. Sequencing results depicting the deleted region in HCT116$^{YY2null}$ cells were shown. **(D)** YY2 and YY1 protein expression levels in HCT116$^{YY2null}$ cells, as determined using western blotting. **(E)** YY2 protein expression level in HCT116 cells stably overexpressing *YY2* established using lentivirus, as determined using western blotting. Wild-type HCT116 cells, HCT116 cells transfected with shCon or pcCon, or infected with empty lentivirus (EV) were used as controls. β-actin was used for western blotting loading control. Quantification data are shown as mean ± SD (n = 3). *P* values were calculated using two-tailed unpaired Student's t-test. pcCon: pcDNA3.1(+); YY2KO: HCT116$^{YY2null}$ cells; $^{**}P < 0.01$.

# Figure S3.

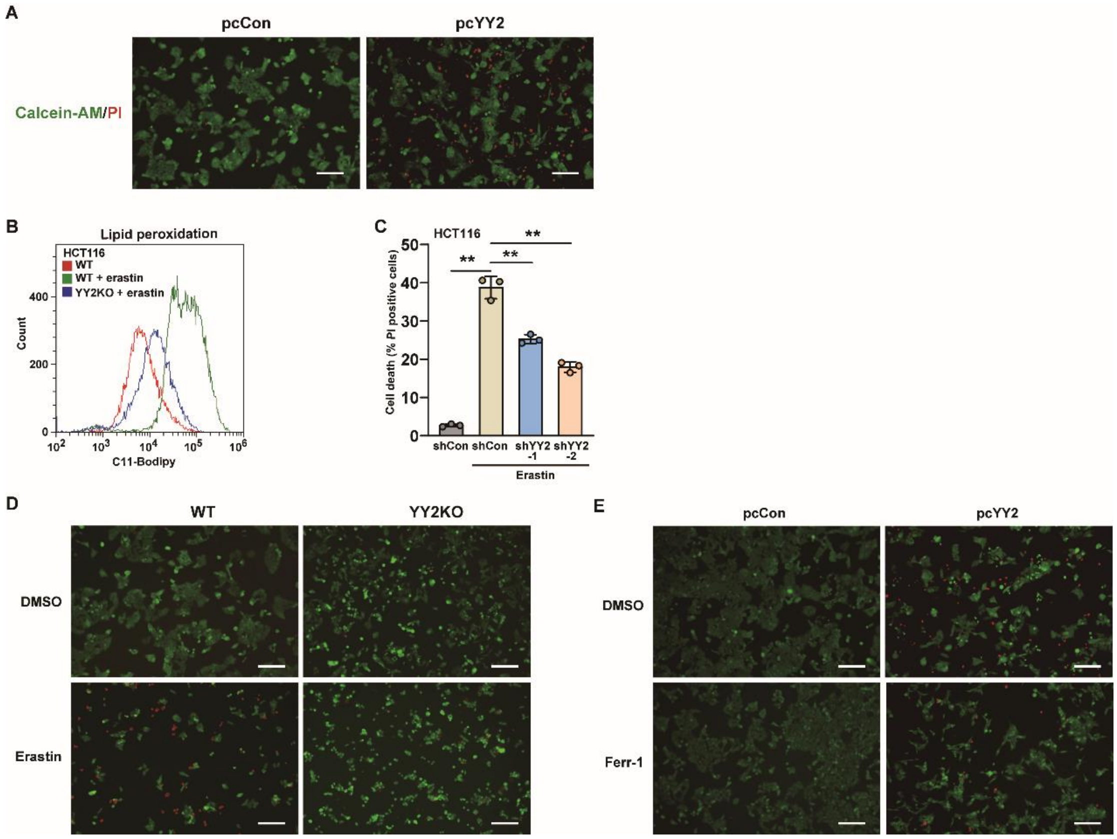


**Supplementary Fig. S3. YY2 regulates tumor cells ferroptotic cell death. (A)** Staining of living cells (calcein-AM, green) and dead cells (PI, red) in HCT116 cells overexpressing *YY2*. Scale bars, 100 μm. **(B)** Lipid peroxidation level in HCT116$^{YY2null}$ cells treated with 20 μM erastin for 24 h, as assessed by C11-BODIPY staining and flow cytometry. **(C)** Cell death percentage of *YY2*-knocked down HCT116 cells treated with 20 μM erastin for 24 h, as determined using PI staining and flow cytometry. **(D)** Staining of living and dead cells in HCT116$^{YY2null}$ cells treated with 20 μM erastin for 24 h using calcein-AM/PI. Scale bars, 100 μm. **(E)** Staining of living and dead cells in HCT116 cells overexpressing *YY2* and treated with 30 μM ferrostatin-1 for 24 h using calcein-AMP/PI. Scale bars, 100 μm. Wild-type HCT116 cells or HCT116 cells transfected with shCon or pcCon were used as controls. Quantification data are shown as mean ± SD (n = 3). *P* values were calculated using two-tailed unpaired Student's t-test. One-way ANOVA analyses were performed when more than two groups were compared. pcCon: pcDNA3.1(+); YY2KO: HCT116$^{YY2null}$ cells; Ferr-1: ferrostatin 1; $**P < 0.01$.

## Figure S4.

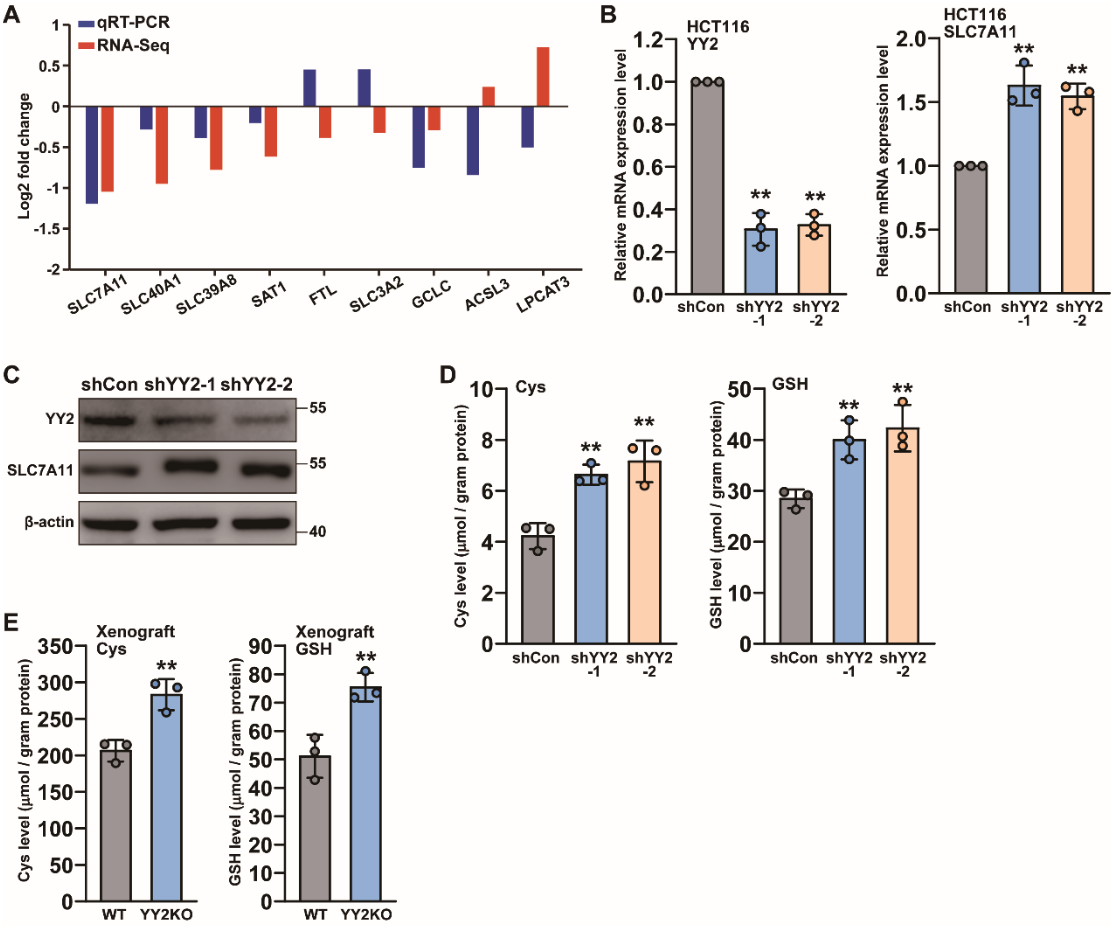


**Supplementary Fig. S4. *YY2*-silencing suppresses SLC7A11 and induces cellular cysteine and GSH levels. (A)** Fold-change of mRNA expression levels of candidate target genes related to ferroptosis in HCT116 overexpressing *YY2* compared to HCT116 cells transfected with pcCon, as determined using qRT-PCR. **(B and C)** YY2 and SLC7A11 mRNA (B) and protein (C) expression levels in *YY2*-knocked down HCT116 cells, as determined using qRT-PCR and western blotting, respectively. **(D)** Cysteine (left) and GSH (right) levels in *YY2*-knocked down HCT116 cells. **(E)** Cysteine (left) and GSH (right) levels in xenografted tumors formed by wild-type HCT116 and HCT116$^{YY2null}$ cells. Wild-type HCT116 cells or HCT116 cells transfected with shCon or pcCon were used as controls. β-actin was used for qRT-PCR normalization and as western blotting loading control. Quantification data are shown as mean ± SD (n = 3). *P* values were calculated using two-tailed unpaired Student's t-test. pcCon: pcDNA3.1(+); YY2KO: HCT116$^{YY2null}$ cells; $^{**}P < 0.01$.

**Figure S5.**

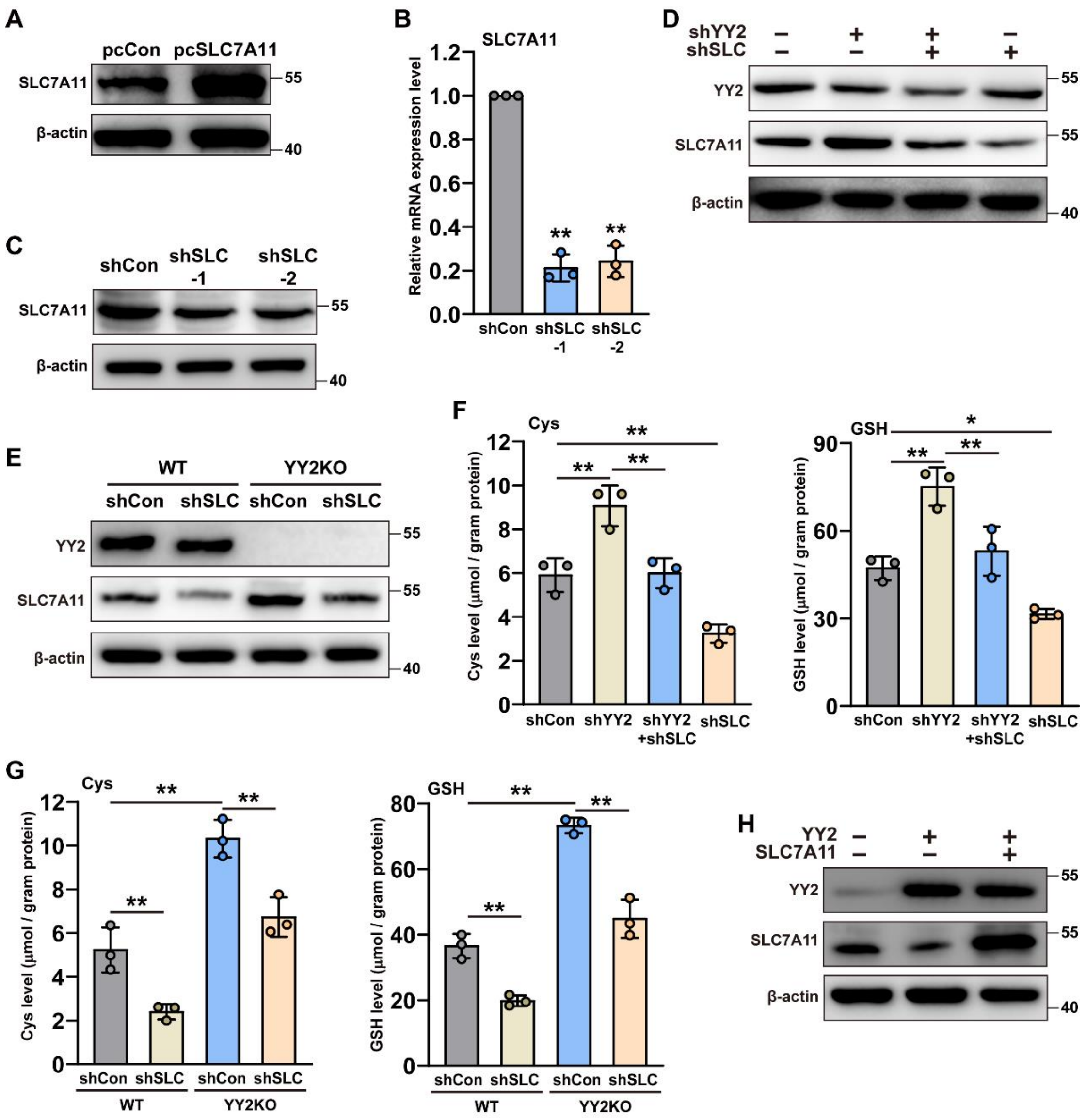


**Supplementary Fig. S5. SLC7A11 is crucial for YY2 suppression on cellular cysteine and GSH levels. (A)** SLC7A11 protein expression level in HCT116 cells transfected with *SLC7A11* overexpression vector, as determined using western blotting. **(B and C)** SLC7A11 mRNA (B) and protein (C) expression levels in HCT116 cells transfected with shRNAs targeting *SLC7A11*, as determined using qRT-PCR and western blotting, respectively. **(D and E)** YY2 and SLC7A11 protein expression levels in *YY2*/*SLC7A11*-double knocked down (D) HCT116 and *SLC7A11*-knocked down HCT116$^{YY2null}$ cells, as examined using western blotting. **(F and G)** Cysteine (left) and GSH (right) levels in *YY2*/*SLC7A11*-double knocked down HCT116 (F) and *SLC7A11*-knocked down HCT116$^{YY2null}$ cells (G). **(H)** YY2 and

SLC7A11 protein expression levels in HCT116 cells infected with Lenti-YY2 (YY2) and Lenti-SLC7A11 (SLC7A11), as determined using western blotting. Wild-type HCT116 cells, HCT116 cells transfected with shCon or pcCon, or infected with empty lentivirus were used as controls. β-actin was used for qRT-PCR normalization and as western blotting loading control. Quantification data are shown as mean ± SD (n = 3). *P* values were calculated using two-tailed unpaired Student's t-test. One-way ANOVA analyses were performed when more than two groups were compared. pcCon: pcDNA3.1(+); YY2KO: HCT116$^{p53null}$ cells; *$P < 0.05$; **$P < 0.01$.

## Figure S6.

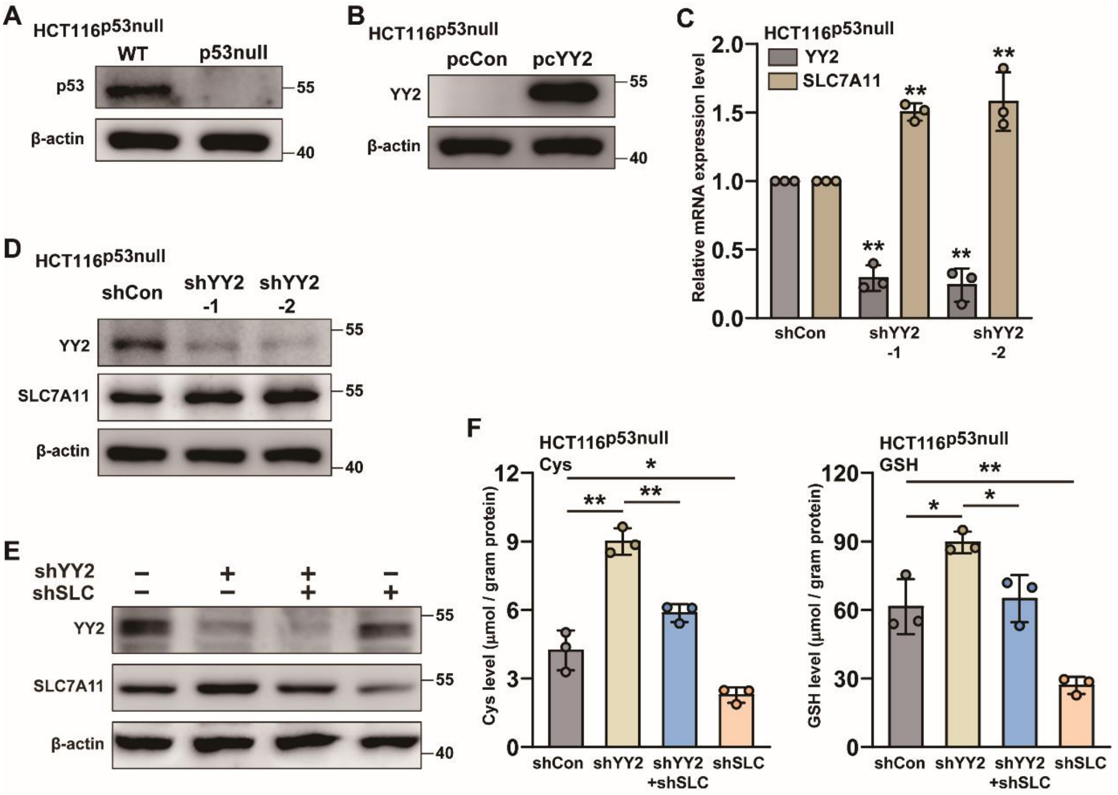


**Supplementary Fig. S6. YY2 regulates SLC7A11/GSH synthesis axis in a p53-independent manner. (A)** p53 protein expression level in HCT116$^{p53null}$ cells, as determined using western blotting. **(B)** YY2 protein expression level in HCT116 $^{p53null}$ cells transfected with *YY2* overexpression vector, as determined using western blotting. **(C and D)** YY2 and SLC7A11 mRNA (C) and protein (D) expression levels in *YY2*-knocked down HCT116$^{p53null}$ cells, as determined using qRT-PCR and western blotting, respectively. **(E)** YY2 and SLC7A11 protein expression levels in *YY2*/*SLC7A11*-double knocked down HCT116$^{p53null}$ cells, as examined using western blotting. **(F)** Cysteine (left) and GSH (right) levels in *YY2*/*SLC7A11*-double knocked down HCT116$^{p53null}$ cells. Cells transfected with shCon or pcCon were used as controls. β-actin was used for qRT-PCR normalization and as western blotting loading control. Quantification data are shown as mean ± SD (n = 3). *P* values were calculated using two-tailed unpaired Student's t-test. One-way ANOVA analyses were performed when more than two groups were compared. pcCon: pcDNA3.1(+); *$P < 0.05$; **$P < 0.01$.

## Figure S7.

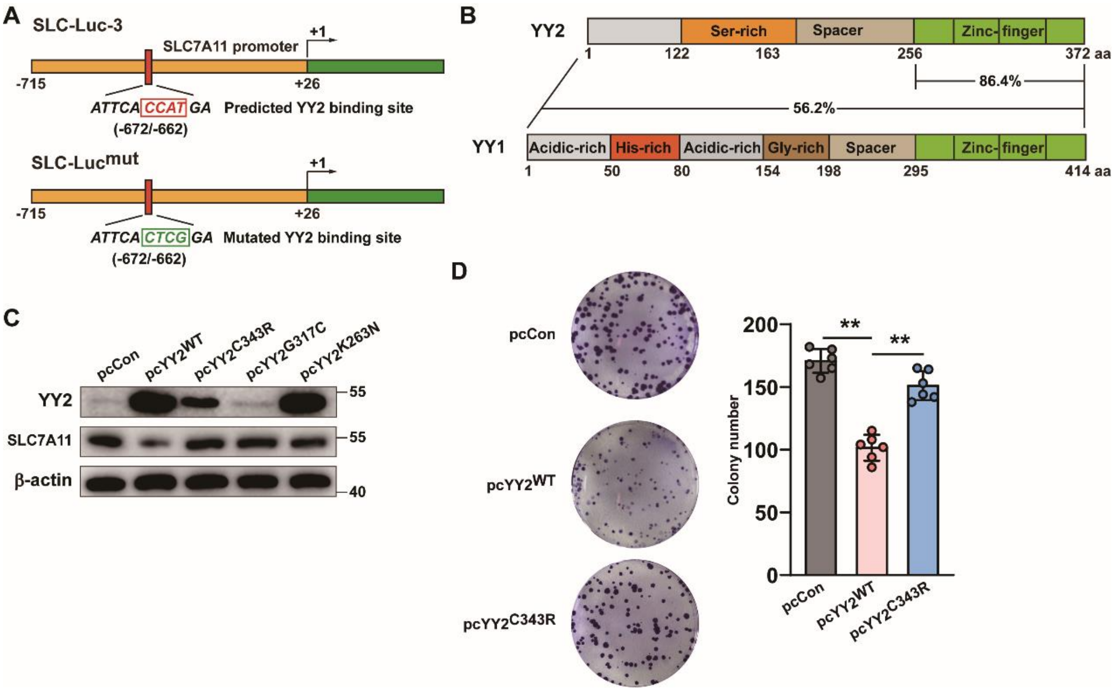


**Supplementary Fig. S7. Mutation in zinc finger domains abolished YY2 regulatory effect on SLC7A11 expression. (A)** Schematic diagram of reporter vector bringing the -715 to +26 region of *SLC7A11*-promoter (SLC-Luc-3; upper panel) and reporter vector bringing the -715 to +26 region of *SLC7A11*-promoter with mutated YY2 predicted binding site (SLC-Luc$^{mut}$; lower panel). The mutated nucleotides are shown in red. **(B)** Schematic diagram showing the comparison between the protein structures of YY2 and YY1. Percentages of identity between the whole proteins and the zinc-fingers domains are shown. **(C)** SLC7A11 protein expression level in HCT116 cells overexpressing cancer-associated *YY2* mutants and wild-type *YY2* (WT), as determined using western blotting. **(D)** Colony formation potential of HCT116 cells overexpressing *YY2$^{C343R}$* mutant. Representative images and quantification results (n = 6) are show. Cells transfected with pcCon or wild-type *YY2* overexpression vector were used as controls. β-actin was used for western blotting loading control. Quantification data are shown as mean ± SD. *P* values were calculated using one-way ANOVA analyses. pcCon: pcDNA3.1(+); $^{**}P < 0.01$.

## Figure S8.

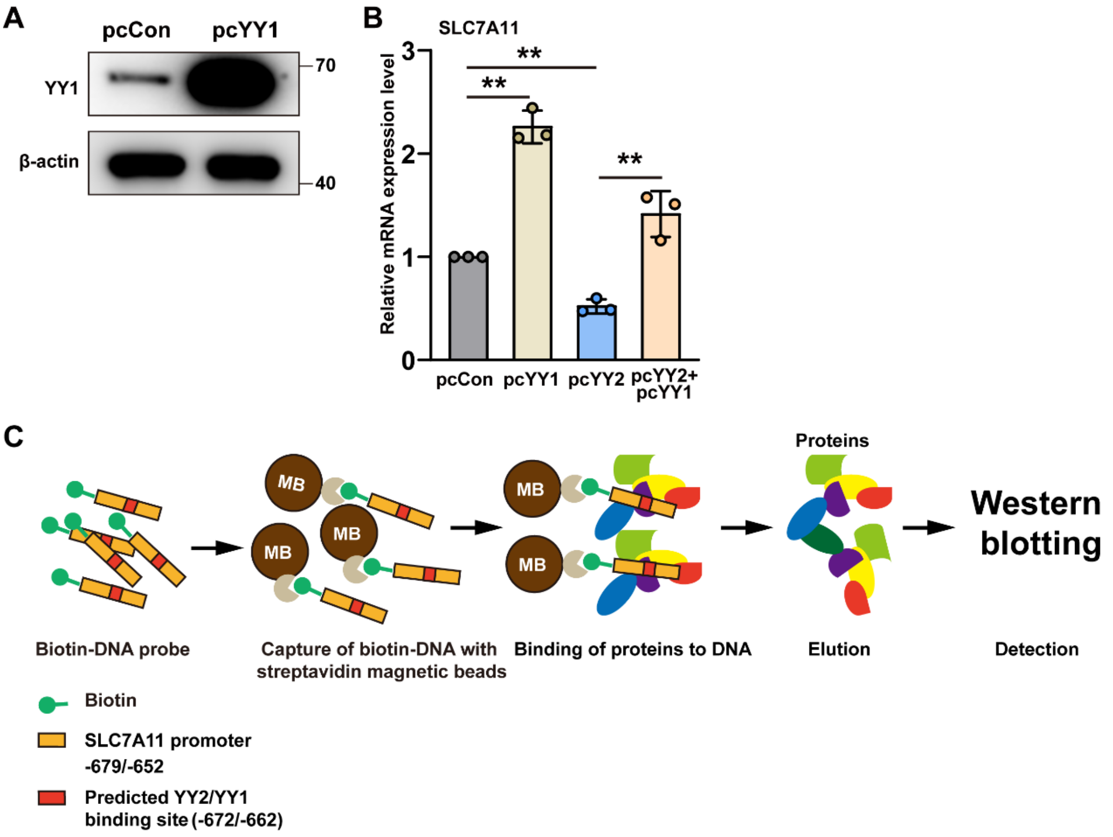


**Supplementary Fig. S8. YY2 and YY1 regulate SLC7A11 expression level. (A)** YY1 protein level in HCT116 cells transfected with pcYY1, as determined by western blotting. **(B)** SLC7A11 mRNA expression in HCT116 cells overexpressing *YY2* and/or *YY1*, as determined using qRT-PCR. β-actin was used for qRT-PCR normalization control and as western blotting loading control. **(C)** Schematic diagram of DNA affinity precipitation assay using biotin-DNA probe targeting the -672 to -662 region of the *SLC7A11* promoter. Quantification data are shown as mean ± SD (n = 3). *P* values were calculated using one-way ANOVA analyses. pcCon: pcDNA3.1(+); ***P* < 0.01.

# Figure S9.

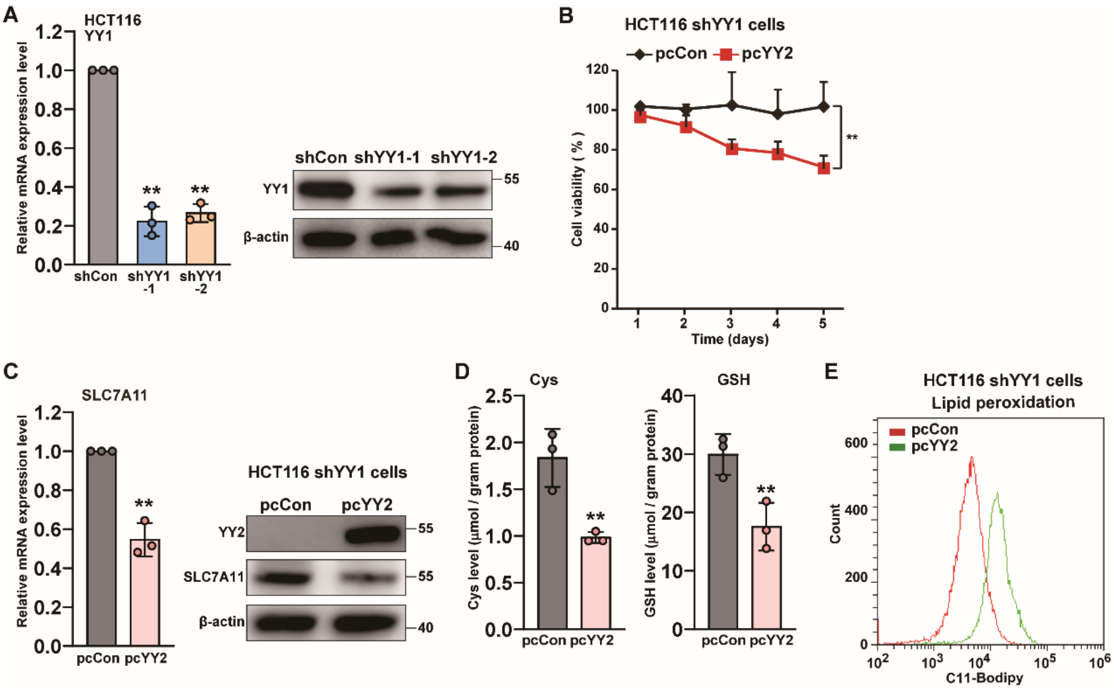


**Supplementary Fig. S9. YY2 regulates SLC7A11/GSH synthesis axis in *YY1*-knocked down cells. (A)** YY1 mRNA (left pannel) and protein (right pannel) expression levels in *YY1*-knocked down HCT116 stable cell lines, as determined using qRT-PCR and western blotting, respectively. **(B)** Viabilities of *YY1*-knocked down HCT116 cells overexpressing *YY2* were measured at indicated time points. **(C)** SLC7A11 mRNA (left) and protein (right) expression levels in *YY1*-knocked down HCT116 cells overexpressing *YY2*, as determined using qRT-PCR and western blotting, respectively. **(D)** Cysteine (left) and GSH (right) levels in *YY1*-knocked down HCT116 cells overexpressing *YY2*. **(E)** Lipid peroxidation in *YY1*-knocked down HCT116 cells overexpressing *YY2*, as assessed by C11-BODIPY staining and flow cytometry. Cells transfected with shCon or pcCon were used as controls. β-actin was used for qRT-PCR normalization and as western blotting loading control. Quantification data are shown as mean ± SD (n = 3). *P* values were calculated using two-tailed unpaired Student's t-test. pcCon: pcDNA3.1(+); $**P < 0.01$.

## Figure S10.

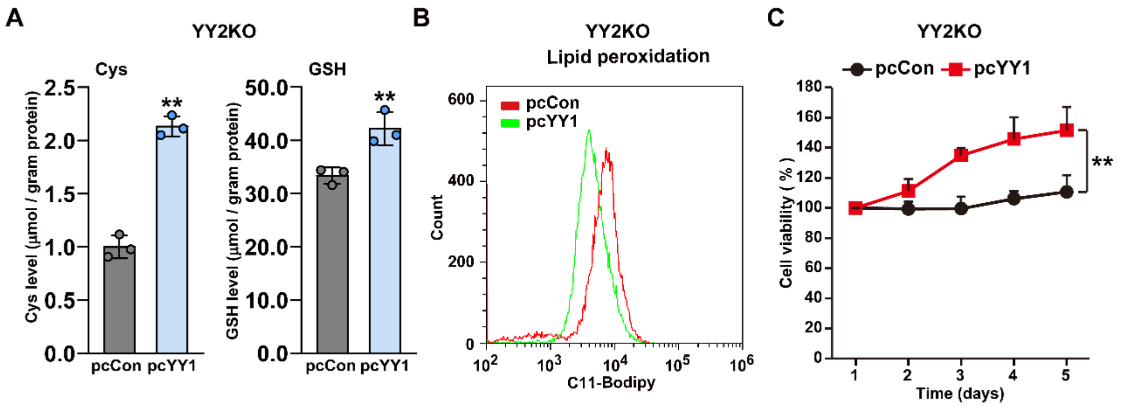


**Supplementary Fig. S10. YY1 regulates ferroptosis in HCT116$^{YY2null}$ cells.** (**A**) Cysteine (left) and GSH (right) levels in HCT116$^{YY2null}$ cells overexpressing *YY1*. (**B**) Lipid peroxidation in HCT116$^{YY2null}$ cells overexpressing *YY1*, as assessed by C11-BODIPY staining and flow cytometry. **(C)** Viabilities of HCT116$^{YY2null}$ cells overexpressing *YY1* were measured at indicated time points. Cells transfected with pcCon were used as controls. Quantification data are shown as mean ± SD (n = 3). *P* values were calculated using two-tailed unpaired Student's t-test. pcCon: pcDNA3.1(+); YY2KO: HCT116$^{YY2null}$ cells; $^{**}P < 0.01$.

**Figure S11.**

**A**

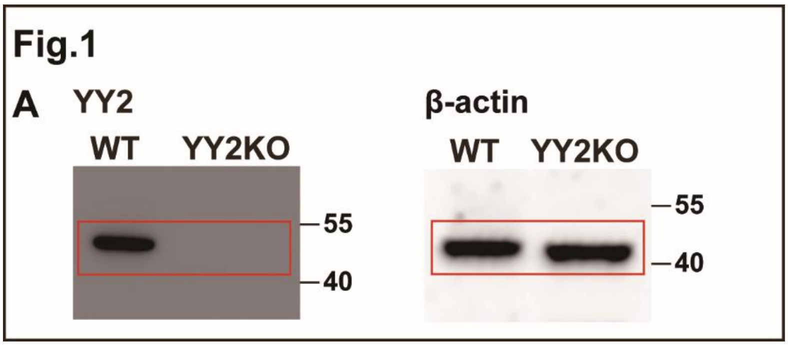


**B**

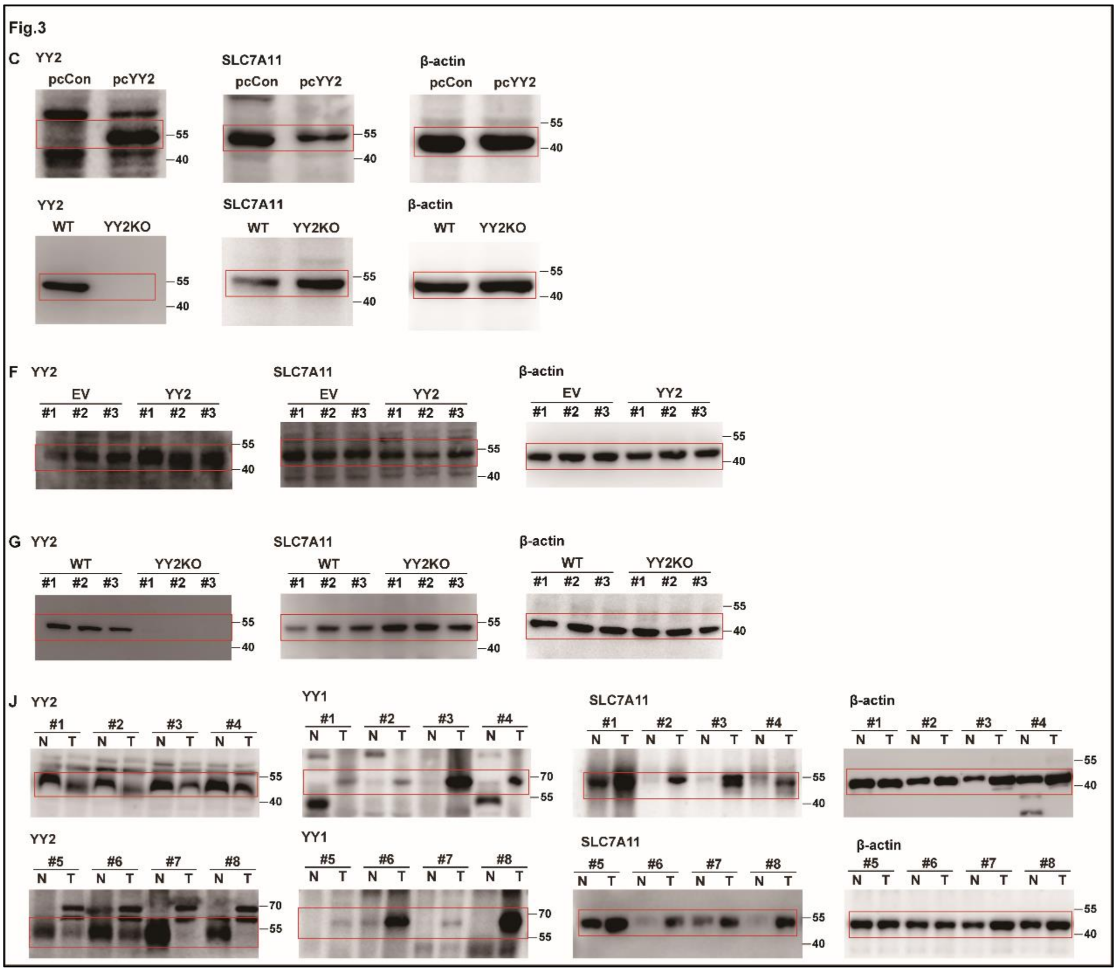


**Supplementary Fig. S11. Uncropped western blots with the indicated areas of selection in Figures 1, 3 to 6 and 8, Figures S1, S2, and S4 to S9.** (continued)

C

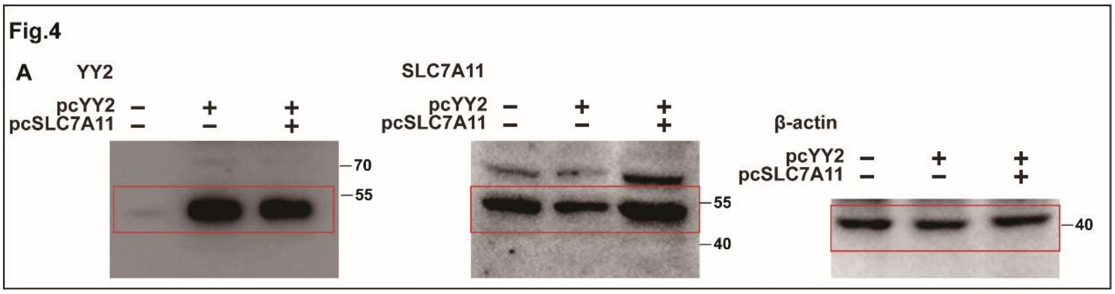


D

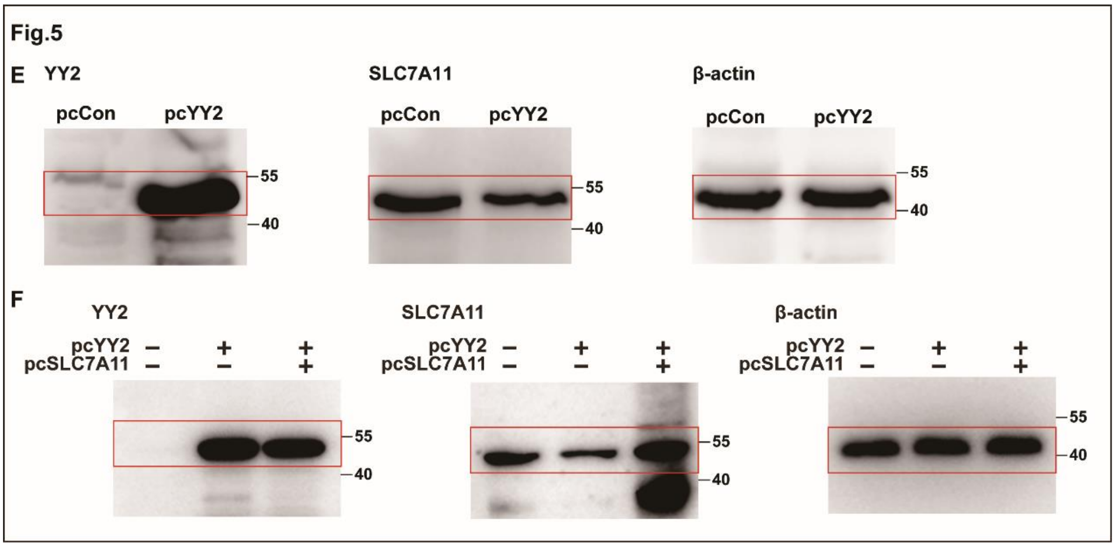


E

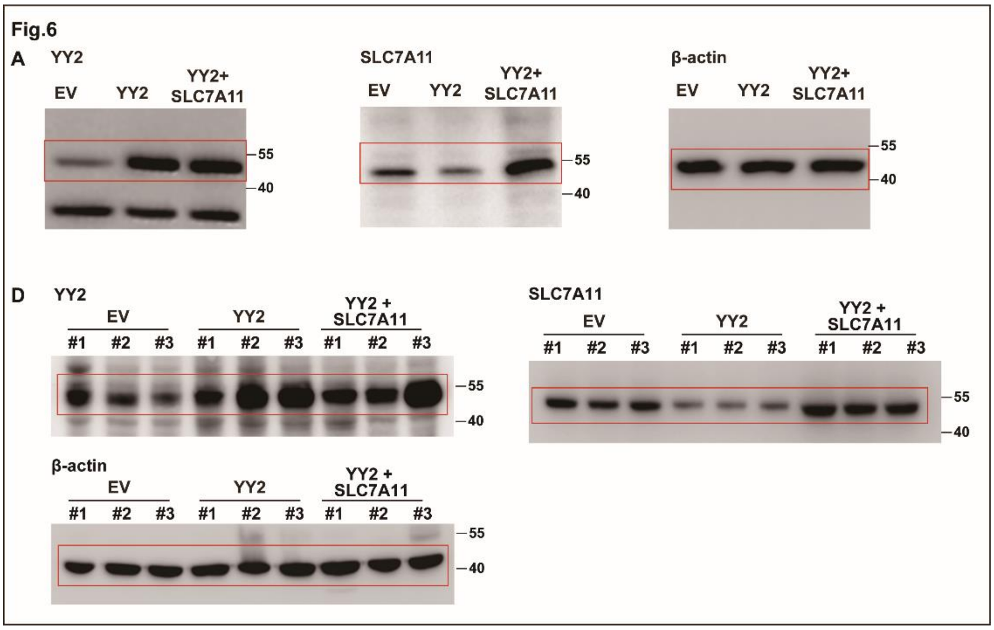


**Supplementary Fig. S11. Uncropped western blots with the indicated areas of selection in Figures 1, 3 to 6 and 8, Figures S1, S2, and S4 to S9.** (continued)

**F**

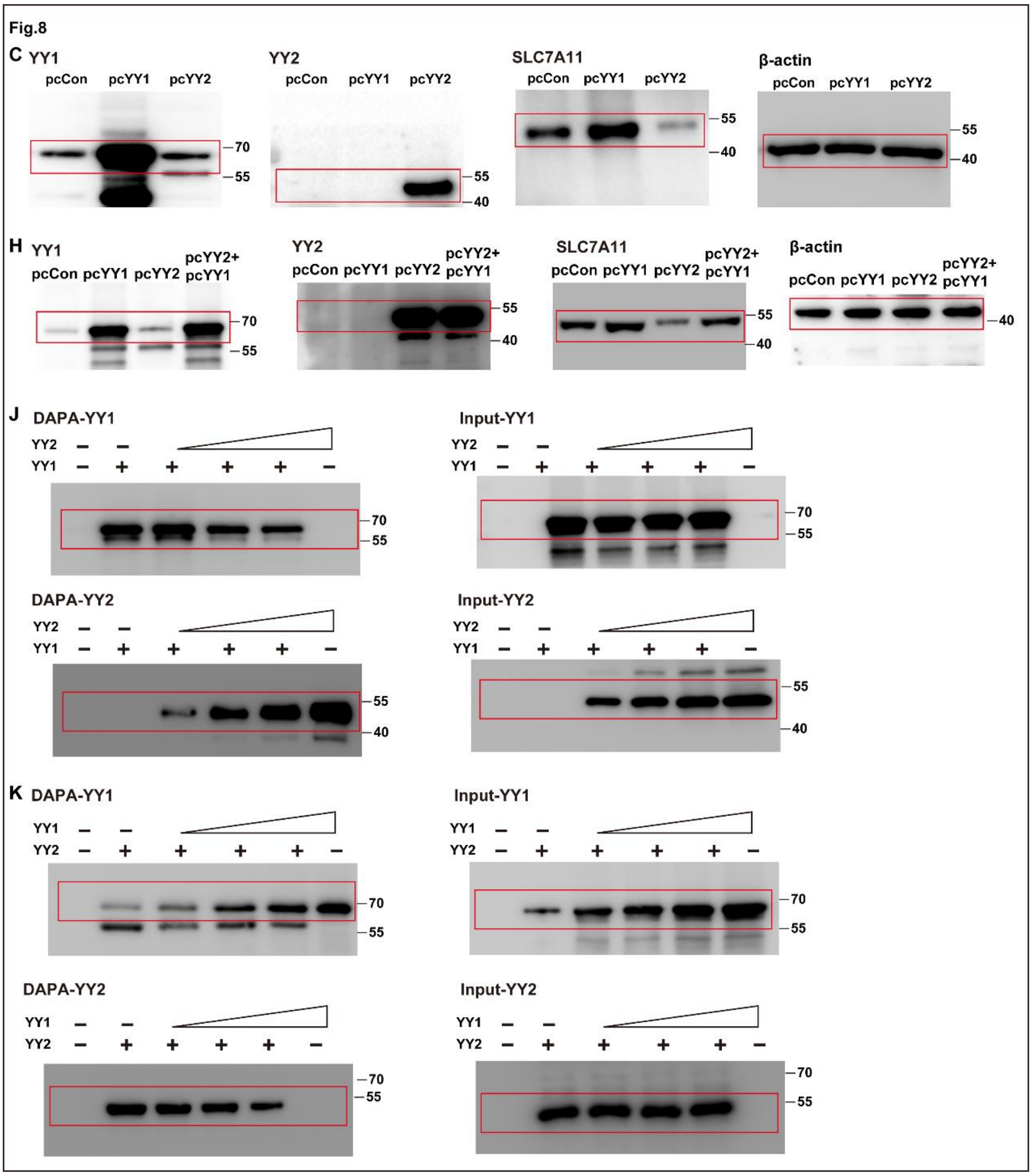


**Supplementary Fig. S11. Uncropped western blots with the indicated areas of selection in Figures 1, 3 to 6 and 8, Figures S1, S2, and S4 to S9.** (continued)

**G**

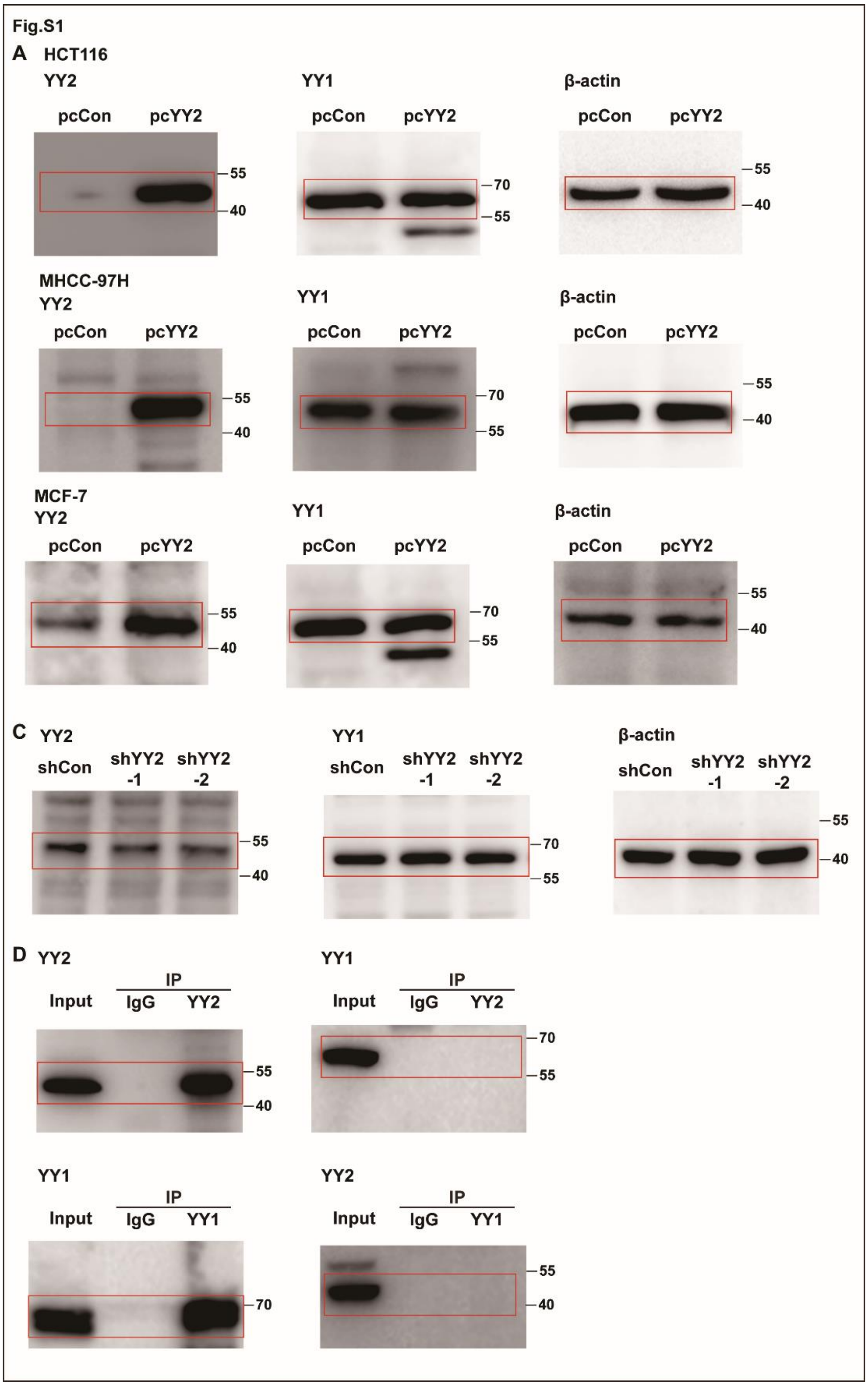


**Supplementary Fig. S11. Uncropped western blots with the indicated areas of selection in Figures 1, 3 to 6 and 8, Figures S1, S2, and S4 to S9.** (continued)

H

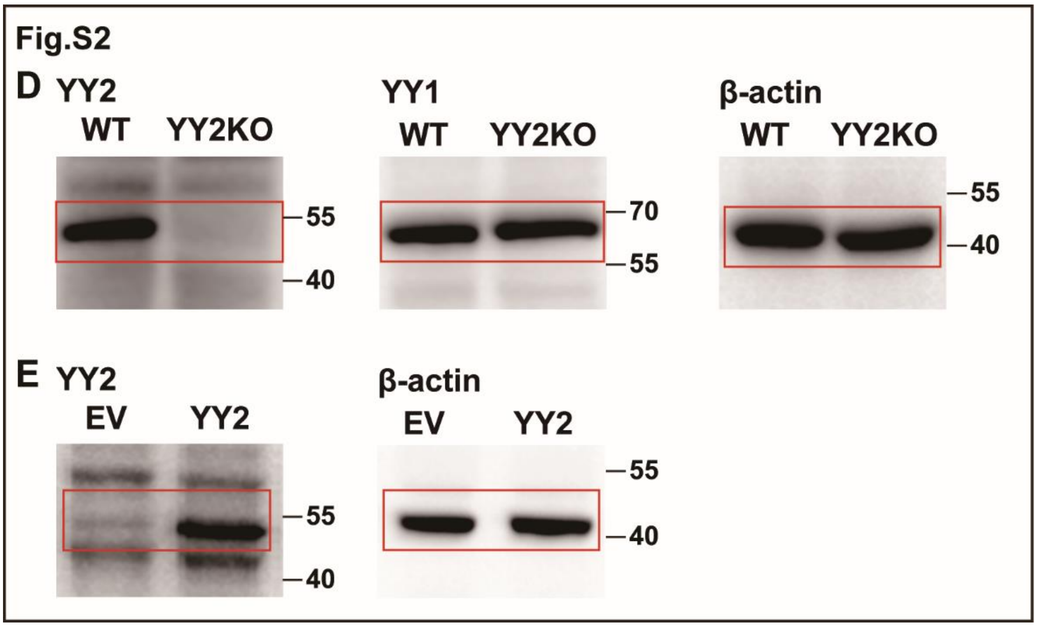


I

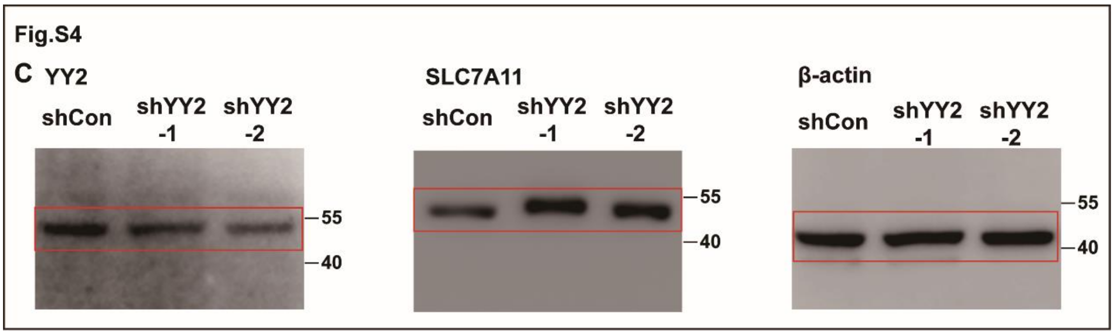


**Supplementary Fig. S11. Uncropped western blots with the indicated areas of selection in Figures 1, 3 to 6 and 8, Figures S1, S2, and S4 to S9.** (continued)

J

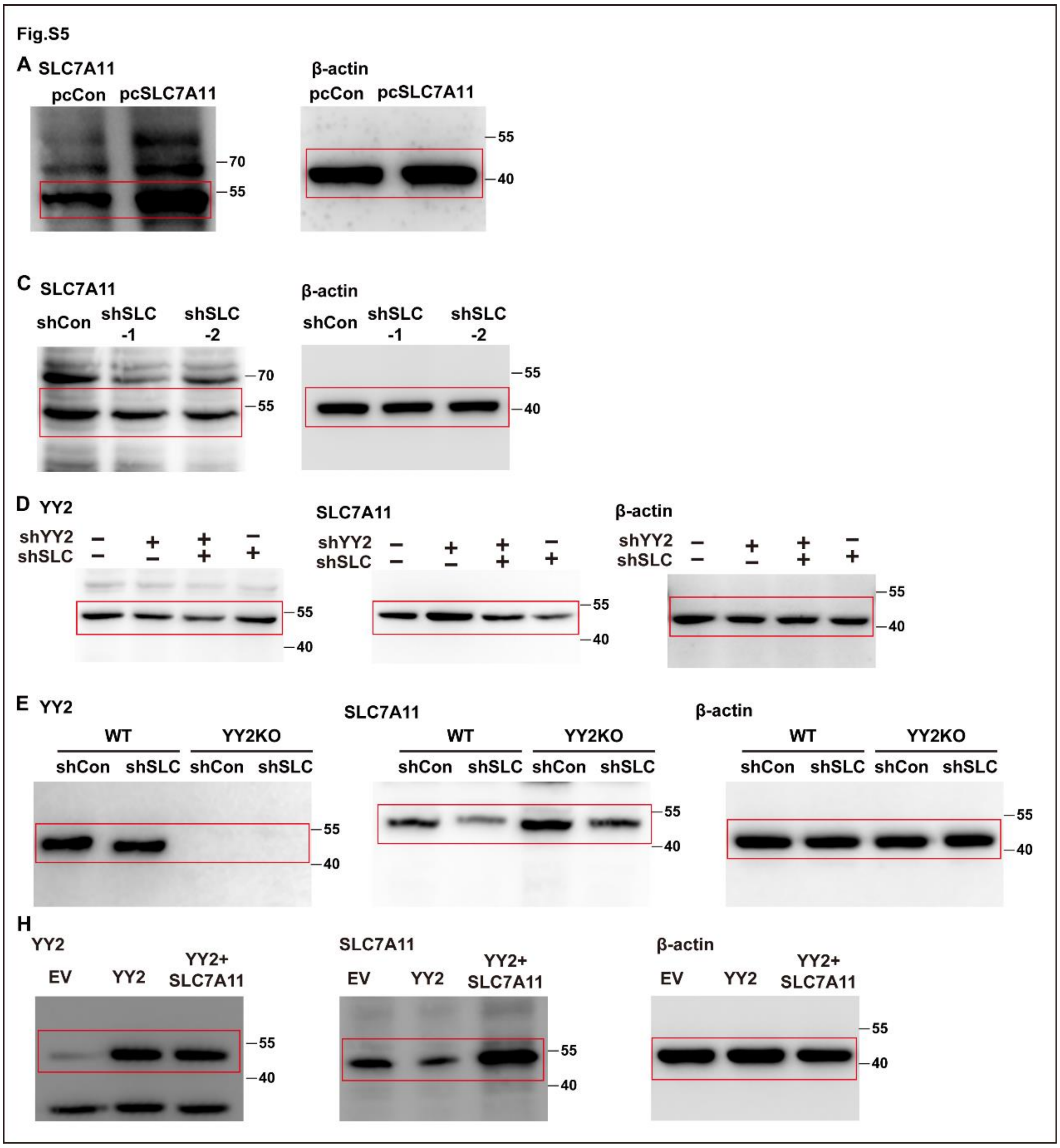


**Supplementary Fig. S11. Uncropped western blots with the indicated areas of selection in Figures 1, 3 to 6 and 8, Figures S1, S2, and S4 to S9.** (continued)

**K**

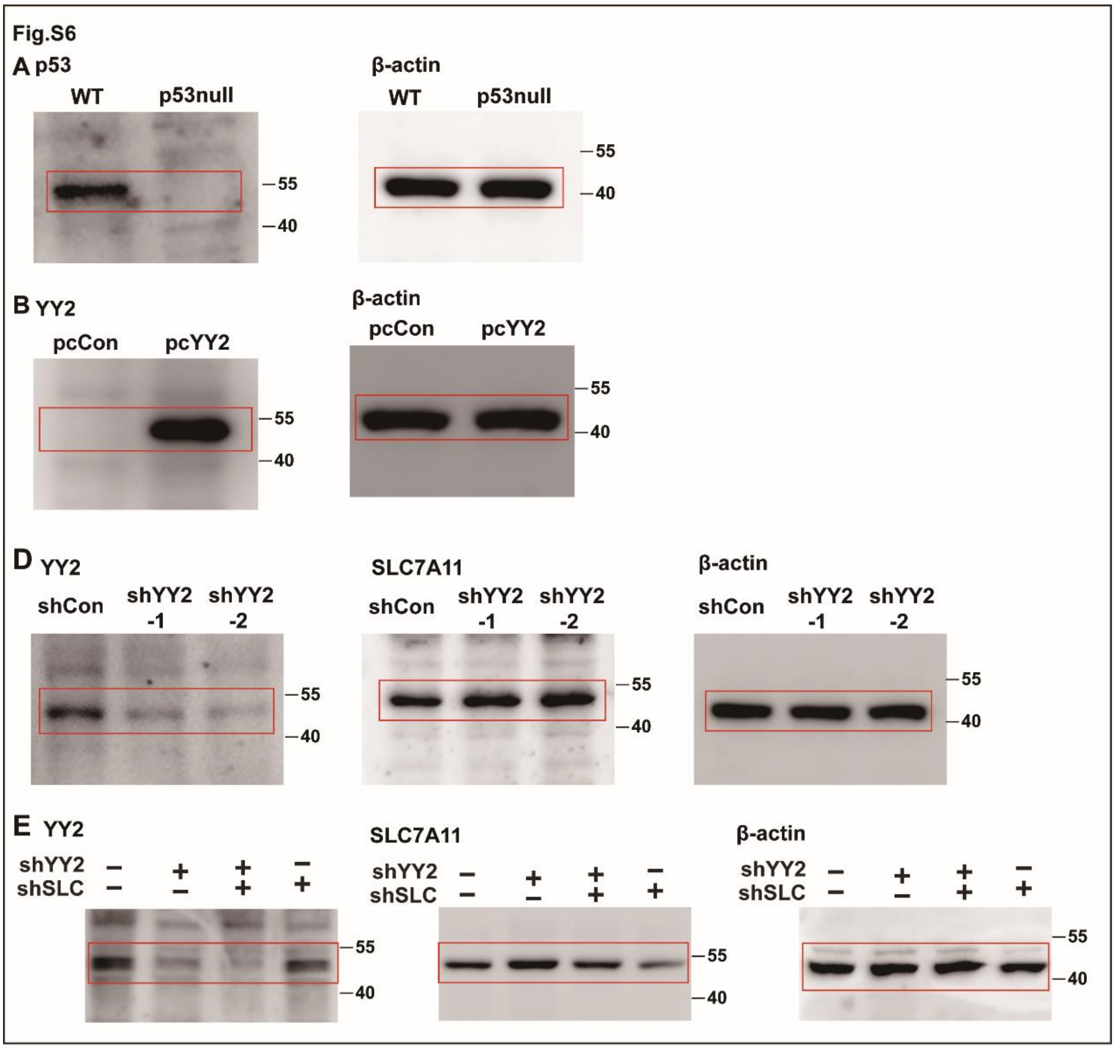


**Supplementary Fig. S11. Uncropped western blots with the indicated areas of selection in Figures 1, 3 to 6 and 8, Figures S1, S2, and S4 to S9.** (continued)

**L**

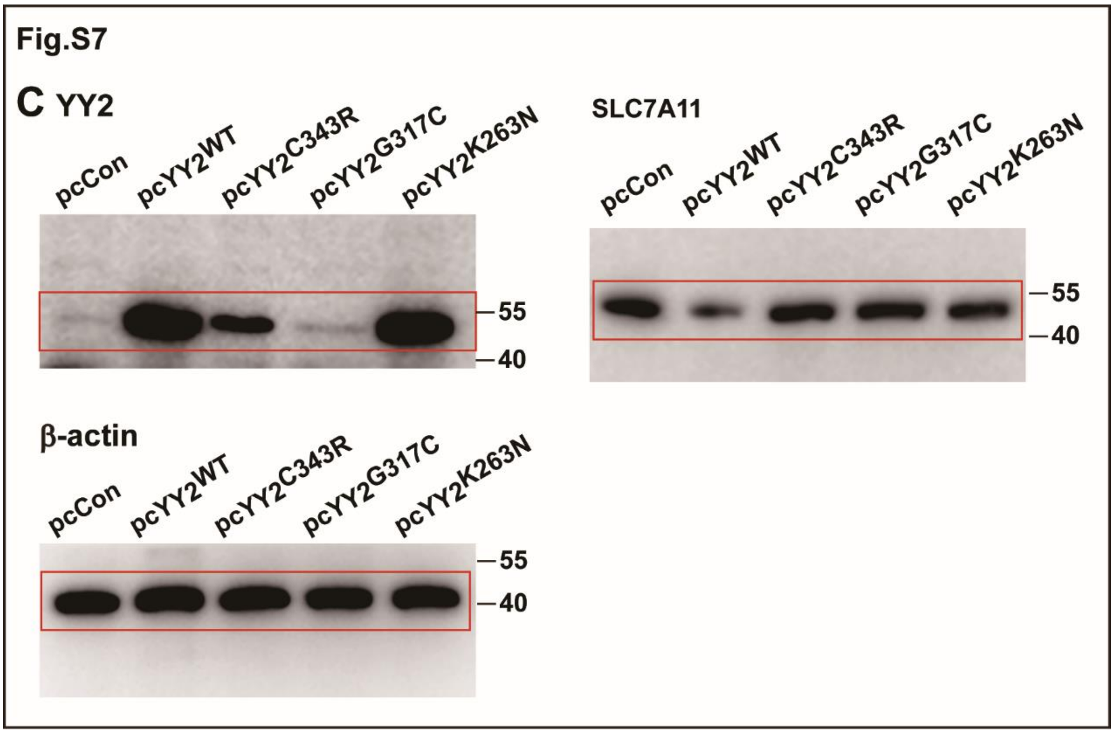


**M**

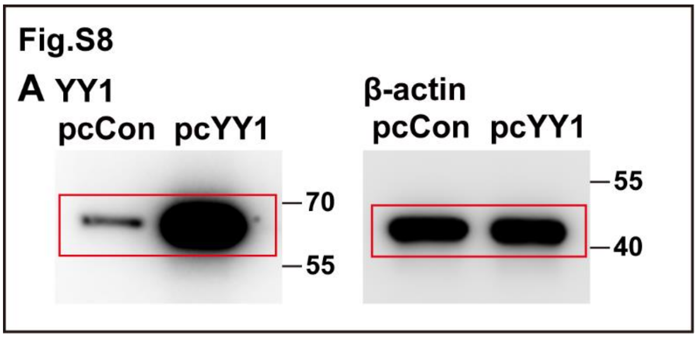


**Supplementary Fig. S11. Uncropped western blots with the indicated areas of selection in Figures 1, 3 to 6 and 8, Figures S1, S2, and S4 to S9.** (continued)

**N**

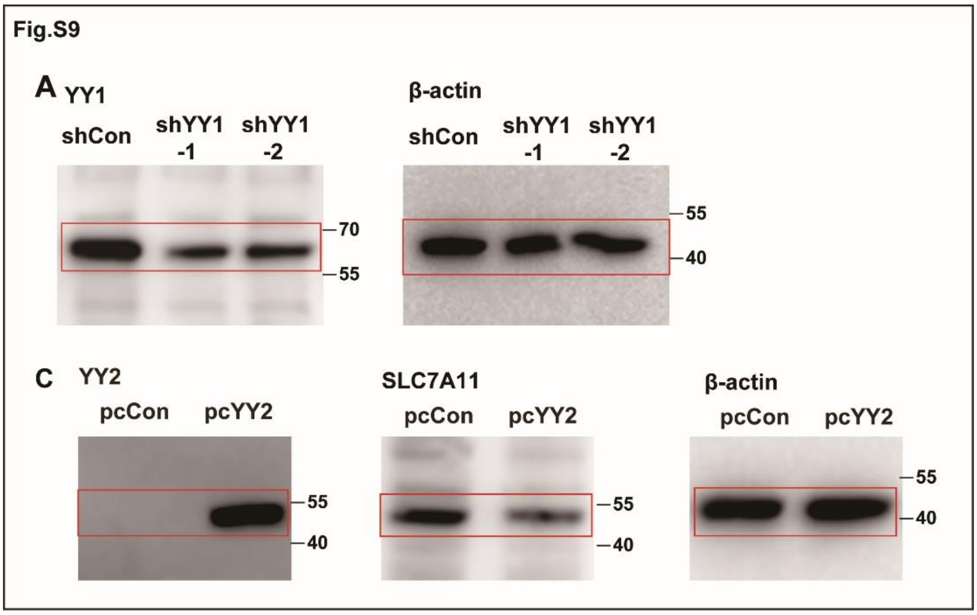


**Supplementary Fig. S11. Uncropped western blots with the indicated areas of selection in Figures 1, 3 to 6 and 8, Figures S1, S2, and S4 to S9.** (continued)

**Table S1. Primer pairs used for gene quantification by quantitative RT-PCR.**

| **Genes** | **Refseq No.** | **Forward primer sequence (5′-3′)** | **Reverse primer sequence (5′-3′)** |
|---|---|---|---|
| YY2 | NM_206923.4 | GAAGTGGTGGGCTATTGCGA | AGGGTCATCTGGAAGTGCTC |
| YY1 | NM_003403.4 | GCGGAGCCCTCAGCCATGGCCTCG | CAGCGGCTGCAGAGCGATCATGG |
| SLC7A11 | NM_014331.4 | TCCTGCTTTGGCTCCATGAACG | AGAGGAGTGTGCTTGCGGACAT |
| SLC40A1 | NM_014585.6 | CAGTTTGCAACATGTCTGTACC | TAGCAACGTATTGCAGTCTCC |
| SLC39A8 | NM_001135147.1 | CTGCAATACCAGCACCTTCA | CACACACAGCATCACGTCTC |
| SAT1 | NM_213613.4 | GTCTTTGCCTAACGTCGCTG | TTAGGGAGGCGGTGCTTAGA |
| FTL | NM_000146.4 | CAACCAACCATGAGCTCCCA | TTTCATGGCGTCTGGGGTTT |
| SLC3A2 | NM_002394.6 | GTTTGTCTCAGGCAAGGCTC | GGAACAAGGAAAGGAGGGAG |
| GCLC | NM_001498.4 | GAGGTCAAACCCAACCCAGT | AAGGTACTGAAGCGAGGGTG |
| ACSL3 | NM_004457.5 | TGACACAAGGGCGCATATCT | CCAGTCCTTCCCAACAACGA |
| LCPAT3 | NM_005768.6 | TGTGGAAAGACAGGCTGCCA | GAAGAGGCAGAAGGCAGTCAT |
| β-actin | NM_001101.3 | CGAGCGCGGCTACAGCTT | TCCTTAATGTCACGCACGATTT |

**Table S2. Antibodies used for western blotting, immunohistochemical staining and ChIP assay.**

| **Antibody** | **Product number** | **Maker** | **Experiment** | **Dilution** |
|---|---|---|---|---|
| Anti-YY2 | sc-374455 | Santa Cruz Biotechnology | Western blotting<br>Immunohistochemistry<br>Chromatin Immunoprecipitation<br>Immunoprecipitation | 1/1000<br>1/100<br>30 μg/mL cell lysate<br>25 μg/mL cell lysate |
| Anti-YY1 | 22156-1-AP | Proteintech | Western blotting<br>Immunohistochemistry<br>Chromatin Immunoprecipitation<br>Immunoprecipitation | 1/1000<br>1/200<br>30 μg/mL cell lysate<br>25 μg/mL cell lysate |
| Anti-SLC7A11 | 26864-1-AP | Proteintech | Western blotting<br>Immunohistochemistry | 1/1500<br>1/500 |
| Anti-p53 | 60283-2- Ig | Proteintech | Western blotting | 1/1000 |
| Anti-β-actin | 60008-1- Ig | Proteintech | Western blotting | 1/100000 |
| Anti-histone H3 | 17168-1-AP | Proteintech | Chromatin Immunoprecipitation | 30 μg/mL cell lysate |
| Anti-4-HNE | CABT-B1068 | Creative Diagnostics | Immunohistochemistry | 1/500 |
| Goat anti-rabbit IgG | ZB2301 | ZSGB-BIO | Western blotting | 1/10000 |
| Goat anti-mouse IgG | ZB2305 | ZSGB-BIO | Western blotting | 1/10000 |
